# Validation of Polygenic Risk Scores for Coronary Heart Disease in a Middle Eastern Cohort Using Whole Genome Sequencing

**DOI:** 10.1161/CIRCGEN.122.003712

**Published:** 2022-10-12

**Authors:** Mohamad Saad, Ayman El-Menyar, Khalid Kunji, Ehsan Ullah, Jassim Al Suwaidi, Iftikhar J. Kullo

**Affiliations:** 1 Qatar Computing Research Institute, Hamad Bin Khalifa University, Doha, Qatar (M.S., K.K., E.U.); 2 Hamad Medical Corporation, Doha, Qatar (A.E.-M., J.A.S.); 3 Department of Cardiovascular Medicine, and the Gonda Vascular Center, Mayo Clinic, Rochester, MN (I.J.K.)

**Keywords:** coronary heart disease, diverse populations, Middle East, precision medicine, polygenic risk score, whole genome sequencing

## Abstract

Enthusiasm for using polygenic risk scores (PRSs) in clinical practice is tempered by concerns about their portability to diverse ancestry groups, thus motivating genome-wide association studies in non-European ancestry cohorts.

**Methods:**

We conducted a genome-wide association study for coronary heart disease in a Middle Eastern cohort using whole genome sequencing and assessed the performance of 6 PRSs developed with methods including LDpred (PGS000296), metaGRS (PGS000018), Pruning and Thresholding (PGS000337), and an EnsemblePRS we developed. Additionally, we evaluated the burden of rare variants in lipid genes in cases and controls. Whole genome sequencing at 30× coverage was performed in 1067 coronary heart disease cases (mean age=59 years; 70.3% males) and 6170 controls (mean age=40 years; 43.5% males).

**Results:**

The majority of PRSs performed well; odds ratio (OR) per 1 SD increase (OR_1sd_) was highest for PGS000337 (OR_1sd_=1.81, 95% CI [1.66–1.98], *P*=3.07×10^−41^). EnsemblePRS performed better than individual PRSs (OR_1sd_=1.8, 95% CI [1.66–1.96], *P*=5.89×10^−44^). The OR for the 10th decile versus the remaining deciles was >3.2 for PGS000337, PGS000296, PGS000018, and reached 4.58 for EnsemblePRS. Of 400 known genome-wide significant loci, 33 replicated at *P*<10^−4^. However, the 9p21 locus did not replicate. Six suggestive (*P*<10^−5^) new loci/genes with plausible biological function were identified (eg, *CORO7*, *RBM47*, *PDE4D*). The burden of rare functional variants in *LDLR*, *APOB*, *PCSK9*, and *ANGPTL4* was greater in cases than controls.

**Conclusions:**

Overall, we demonstrate that PRSs derived from European ancestry genome-wide association studies performed well in a Middle Eastern cohort, suggesting these could be used in the clinical setting while ancestry-specific PRSs are developed.

Coronary heart disease (CHD) is the main cause of mortality and morbidity in the world, including in the Middle East (ME).^1^ Lifestyle, environmental, and genetic factors influence predisposition to CHD. Most genetic factors affecting the risk of CHD have been identified using genome-wide association studies (GWAS).^2–9^ To date, more than 260 CHD loci have been identified and generally confer modest risk.^10^ A polygenic risk score (PRS) is an aggregation of numerous modest-effect susceptibility variants and can be integrated with conventional CHD risk factors such as age, sex, blood pressure, and family history of CHD to refine risk estimates for CHD.^11,12^

The majority of CHD GWAS have been conducted in individuals of European ancestry. Enthusiasm for using PRSs in clinical practice is tempered by concerns about their portability to diverse ancestry groups, which remains unclear.^12–14^ An LDpred PRS^14^ and metaGRS^12^ performed well in European and Latino ancestry groups but showed weaker association in African Americans.^13^ The PRSs were validated in a French-Canadian cohort,^15^ and LDpred PRS was also validated in South Asians.^16^

To prevent disparities in genomic medicine, there is an urgent need to conduct GWAS in non-European ancestry cohorts, and enable development of ancestry-specific PRSs and discovery of new susceptibility loci.^13^ The burden of CHD in the ME is rising, likely because of lifestyle patterns and high levels of comorbidities (eg, type 2 diabetes^17^). Little is known about the genetic basis of CHD in the ME.^18,19^ Prior GWAS in ME populations failed to replicate several known CHD loci.^20–22^ To address this gap in knowledge, we initiated the Qatar Cardiovascular Biorepository in 2014 to collect DNA and plasma of CHD cases and controls, with the goal of studying the “omics” of CHD in Qatari individuals.^23^ Whole genome sequencing (WGS) of Qatar Cardiovascular Biorepository CHD participants and a set of controls from the Qatar Biobank (QBB)^24^ was performed in collaboration with the Qatar Genome Program. We assessed the performance of 6 genome-wide PRSs developed mainly in European ancestry cohorts and attempted to replicate known CHD loci and identify new loci. The availability of WGS data was exploited to identify rare pathogenic variants within genes influencing lipoprotein metabolism and subsequently assess the burden of rare variants in cases and controls.

## Methods

Detailed methods are available in the Supplemental Material. The study was approved by the institutional review board, and all participants gave informed consent. Whole-genome sequence data can be accessed through application to QBB/QGP through an established ISO-certified process by submitting a request at (https://www.qatarbiobank.org.qa/research/how-apply), subject to IRB approval by QBB. GWAS summary statistics are available on figshare (https://figshare.com/s/85059db4ddb3a7d27278). The code used to clean the data and run all analyses is available upon request.

## Results

### Cohort Baseline Characteristics

The cohort with WGS consisted of 1035 CHD patients recruited by Qatar Cardiovascular Biorepository and 6202 controls recruited by QBB. Qatari patients with a confirmed diagnosis of CHD or with acute coronary syndrome were approached for participation in the biorepository. Patients were identified at the Heart Hospital Clinic at Hamad Medical Corporation, Qatar. Thirty-two QBB controls reported a history of myocardial infarction in a patient survey and were moved to the case group. After quality control steps (described below), 7023 individuals remained (1014 cases and 6009 controls; 3316 males and 3707 females). Table 1 shows baseline sample characteristics. Age and sex distributions differed between cases (70.3% males, mean age=59±11 years) and controls (43.3% males, mean age=40±12.6 years) with a mean age difference of 19 years. The higher number of males in cases is due to higher prevalence of CHD in males, while among controls, females enrolled more often than males.

**Table 1.**
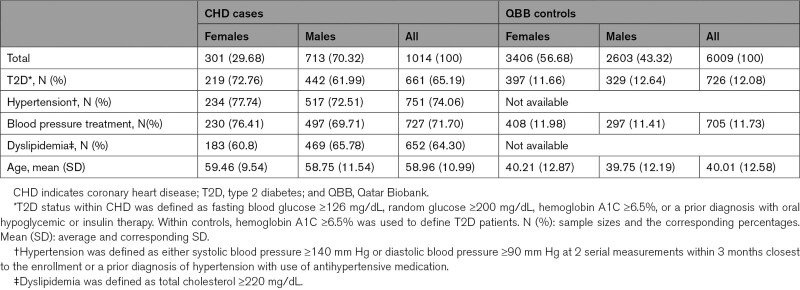
Baseline Characteristics of CHD Cases and Controls After Quality Control Steps

### Ancestry and Admixture

**Figure 1.**
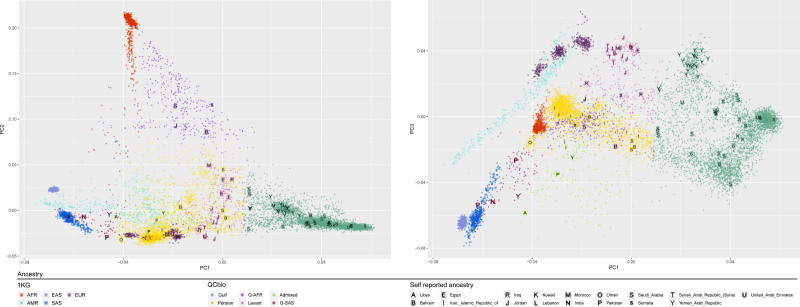
**Principal component analysis of the 6 inferred ancestry groups. A**, PC1 vs PC2; **B**, PC1 vs PC3. Circles represent inferred ancestry groups (Gulf, Persian, Levant, Q-AFR, Admixed, and Q-SAS); Squares represent 1000 Genomes Project ancestry groups. Letters represent the patient self-reported origin/ancestry. PC indicates principal component and QCBio, Qatar Cardiovascular Biorepository.

PC-AiR revealed population stratification and admixture (Figure 1). Participants spanned 6 ancestral groups: Gulf (n=3909, 55.65%), Persian (n=2216, 31.55%), African (Q-AFR, n=343, 4.9%), Levant (n=290, 4.1%), Admixed (n=156, 2.2%), and South Asian (Q-SAS, n=109, 1.6%). Groups were inferred by running PC-AiR on our data and the 1KG data combined,^25^ and leveraging survey information about individual origins in our cohort (Figure 1).

### Polygenic Risk Scores

**Figure 2.**
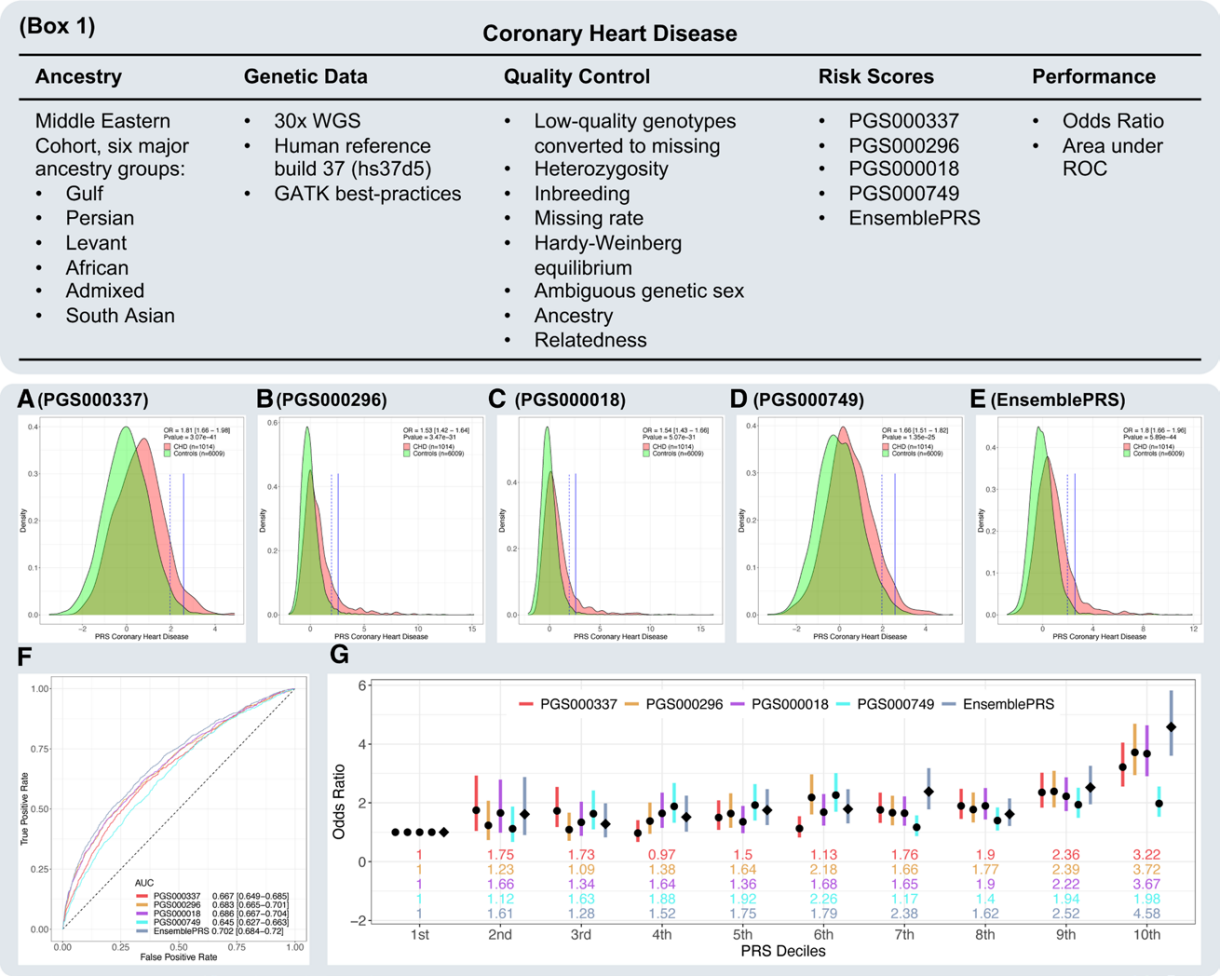
**Polygenic risk score (PRS) reporting scheme recommended by Wand et al.^26^ Box 1**, Information about cohort, data, quality control, and performance metric; (**A–E**) Density plots for PGS000337, PGS000296, PGS000018, PGS000749, and EnsemblePRS. The vertical dotted and continuous lines mark the 0.01 and 0.001 standard normal distribution quantiles; (**F**) AUC; (**G**) Decile plots showing OR_vsAll_. Bars represent the 95% OR_vsAll_ CI. The numbers below the bars are the OR_vsAll_ values. A diamond represents EnsemblePRS. AUC indicates area under the receiver-operator curve; OR, odds ratio; and OR_vsAll_, OR of the 10th decile group vs the remaining groups.

PRS results are shown in Figure 2 and reported following the guidelines in Wand et al.^26^ PGS000116 was dropped due to underperformance (Odds ratio per 1 SD increase [OR_1sd_]=0.87). Based on area under the receiver-operator curve (AUC), PGS000296 and PGS000018 performed similarly (AUC=0.683 [0.665–0.701] and AUC=0.686 [0.667–0.704], respectively; Figure 2F) and outperformed PGS000337 and PGS000749 (AUC=0.667 [0.649–0.685] and AUC=0.645 [0.627–0.663], respectively; Figure 2F). Based on OR_1sd_, PGS000337 (OR_1sd_=1.81 [1.66–1.98], *P*=3.07×10^−41^; Figure 2A) outperformed other PRSs (OR_1sd_=1.53 [1.42–1.64], *P*=3.47×10^−31^ for PGS000296, OR_1sd_=1.54 [1.43–1.66], *P*=5.07×10^−31^ for PGS000018, and OR_1sd_=1.66 [1.51–1.82], *P*=1.35×10^−25^ for PGS000749; Figure 2B through 2D, respectively). EnsemblePRS yielded better predictive power than individual PRSs (OR_1sd_=1.8 [1.66–1.96], AUC=0.702 [0.684–0.72], *P*=5.89×10^−44^; Figure 2E). The AUC of EnsemblePRS was significantly higher than individual PRSs’ AUCs (DeLong’s *P*<5×10^−3^; Supplemental Material). After binning PRSs into 10 deciles, the 10th decile OR_vsAll_ was 1.98 for PGS000749, 3.22 for PGS000337, 3.67 for PGS000018, 3.72 for PGS000296, and 4.58 for the EnsemblePRS (Figure 2G). As expected, the 10th decile OR_vsLowest_ values were larger than their respective OR_vsAll_ (5.77 for PGS000749, 9.27 for PGS000337, 10.1 for PGS000296, 12.37 for PGS000018, and 15.4 for EnsemblePRS Figure S1). Across the ancestry groups in our cohort, PRSs (centered and scaled within each ancestry) generalized relatively well, but results must be interpreted with caution given the small sample sizes for ancestry subgroups, especially among cases. All density plots across ancestries for all PRSs can be found in the Supplemental Material. OR_1sd_, AUC, their 95% CIs, and *P* for all tested models with various combinations of covariates on both the age/sex-matched cohort and the entire cohort are shown in Table S1. As expected, the AUCs for the models that include age, sex, BMI, and 20 PCs were high in the entire cohort (eg, >0.9 for EnsemblePRS) due to the imbalance in age and sex distribution in cases and controls. In the age/sex-matched cohort (1013 out of 6009 controls), AUCs do not vary much across models. The highest AUC was observed for EnsemblePRS for the full model (0.729 [0.714–0.744], *P*=3.58×10^−68^; Table S1).

### Common Variant Association Analyses

#### Discovery Stage

Type 1 error was well controlled with a genomic inflation factor *λ*=1.012, but the statistical distribution was deflated. QQ and Manhattan plots are shown in Figure S2. No single nucleotide variant (SNV) reached genome-wide significance (*P*<5×10^−8^). The most significant SNVs (579 SNVs, *P*<10^−4^) were selected and grouped into independent loci using PLINK’s “--clump” option, yielding 233 loci (Table S2). Only lead SNVs (one per locus) with *P*<10^−5^ were considered for the search of plausible genes (21 SNVs; Table 2). Three SNVs mapped to known CHD genes/loci (within 300 KB of a previously reported SNV in Table S3): *ABCG8* (rs114309202, *P*=3.77×10^−6^), *P4HA2/SLC22A4* (rs75413826, *P*=8.74×10^−6^), and *MIR548A3* (rs17533293, *P*=5.32×10^−6^). Other SNVs mapped to plausible genes: rs917306 (*CORO7/CORO7-PAM16/VASN*, *P*=1.69×10^−6^), rs7690530 (*RBM47*, *P*=3.43×10^−6^), rs12950395 (*HS3ST3B1, P*=9.77×10^−6^), and rs114906338 (*HS3ST1*, *P*=5.45×10^−6^). LocusZoom plots for all 21 lead SNVs are available on Figshare (https://figshare.com/s/579766fd6a6414de599d). Association analysis adjusting for T2D status and the other covariates (ie, 20 PCs, sex, age, and BMI) was performed. Results are shown for the most significant SNVs in Table S2. All top SNVs in Table 2 remained significant, with *P* ranging from 8.02×10^−4^ to 1.56×10^−6^.

**Table 2.**
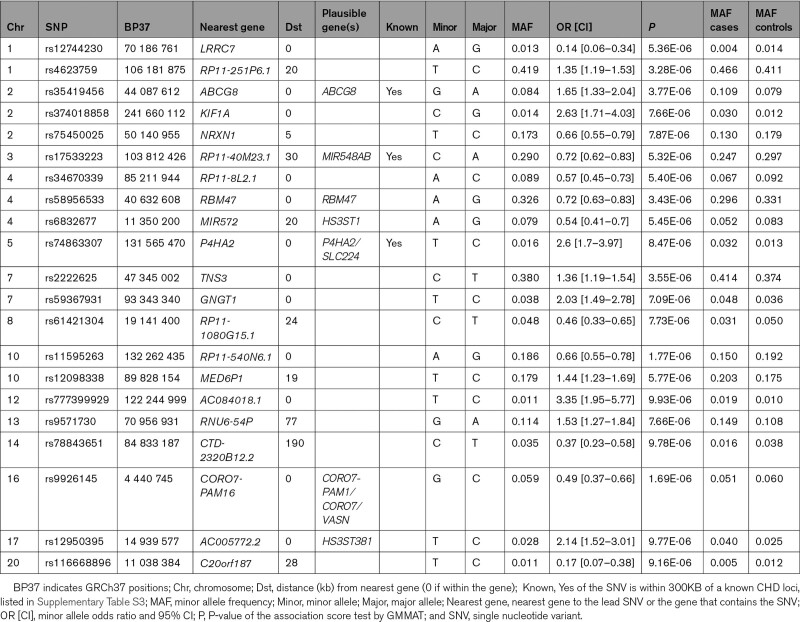
Most Significant Lead SNVs With *P*<10^−5^

### Replication Stage

#### Most Significant SNVs (*P<*10^−4^)

We performed an inverse-variance fixed effect meta-analysis of 9 GWAS summary statistics generated in^2,5,7–9,27^ the FinnGen Release 2 (https://www.finngen.fi/en/access_results; I9_CORATHER), the UKBB (http://pheweb.sph.umich.edu/SAIGE-UKB/pheno/411.4), and the Japan biobank.^28^ Study details are shown in Table S4. There were 6 SNVs with a meta-analysis of *P*<10^−3^ (Table S5): rs7528419 (*CELSR2*, *P*<1×10^−300^), rs114309202 (*ABCG8*, *P*=3.41×10^−6^), rs142264789 (*TRIM5*, *P*=4.58×10^−5^), rs17033109 (*RP13-487K5.1/GUCY1A3*, *P*=1.49×10^−4^), rs62119261 (*APOE*, *P*=1.49×10^−4^), and rs35696698 (*RP11-661D19.3*, *P*=2.12×10^−4^). Five genes/loci have been previously reported for CHD.

#### Known CHD Loci

**Figure 3.**
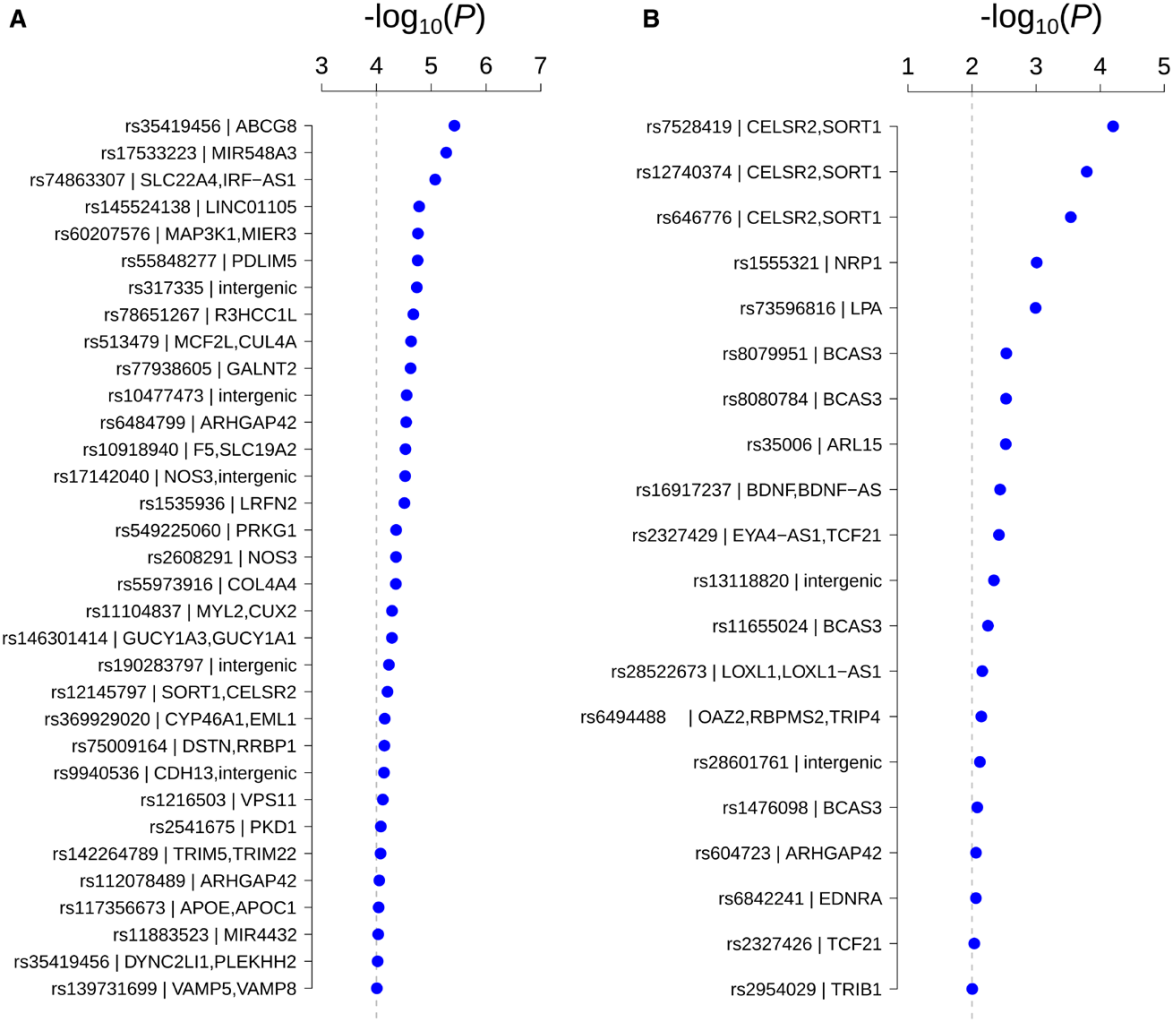
**Replication of known coronary heart disease (CHD) loci. A**, Loci replication: SNVs from our analysis (*P*<10^−4^) that are within 300 KB of a CHD loci; Vertical line is the *P*=10^−4^ cutoff; **B**, Exact match replication: previously reported SNVs replicated in our analysis at *P*<0.01. Vertical line is the *P*=0.01 cutoff.

Replication was assessed for loci and SNVs. A locus was considered to replicate if one of our most significant SNVs was within 300 KB of any of the 936 known SNVs (400 loci). We replicated 33 of the 400 known loci with *P*<10^−4^ (Figure 3A; Tables S2 and S3). The significance of the 771 SNVs that overlapped between the post-QC SNV list and the 936 known SNVs is shown in Table S3. Using a Bonferroni significance level (0.05/771=6.49×10^−5^), only 1 SNV was significant (rs7528419, *SORT1/CELSR2*, *P*=4.82×10^−5^), which is a positive control for our results. At *P*<0.01, the number of significant SNVs was 20 (Figure 3B) and included rs73596816 (*LPA*, *P*=1.02×10^−3^), rs35006 (*ARL15*, *P*=2.98×10^−3^), and rs604723 (*ARHGAP42*, *P*=8.67×10^−3^). The rate of replication was less in other models (with different sets of covariates), although QQplots were not deflated. For instance, in the model that only includes 20 PCs, 883 SNVs (273 loci) had *P*<10^−4^, and only 19 loci of the 400 known loci were replicated (data not shown).

#### 9p21 Locus

**Figure 4.**
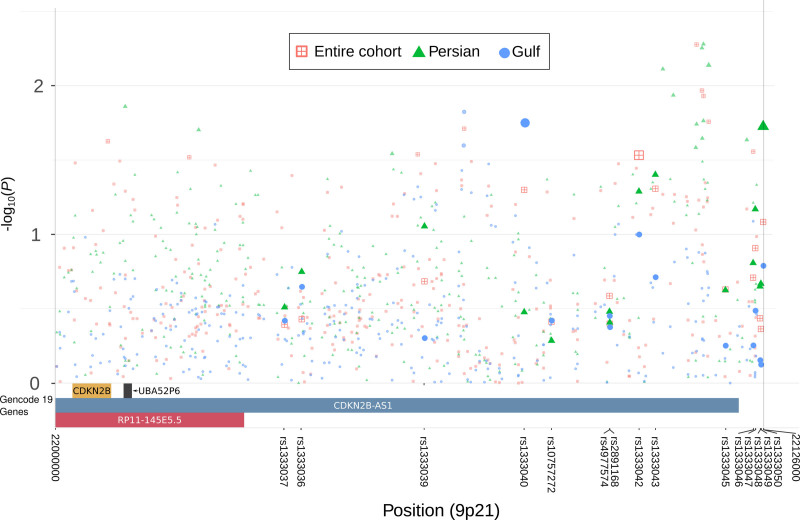
Regional plot for 9p21 showing the 15 selected single nucleotide variants and the significance for the entire cohort, Gulf, and Persian ancestry groups.

**Figure 5.**
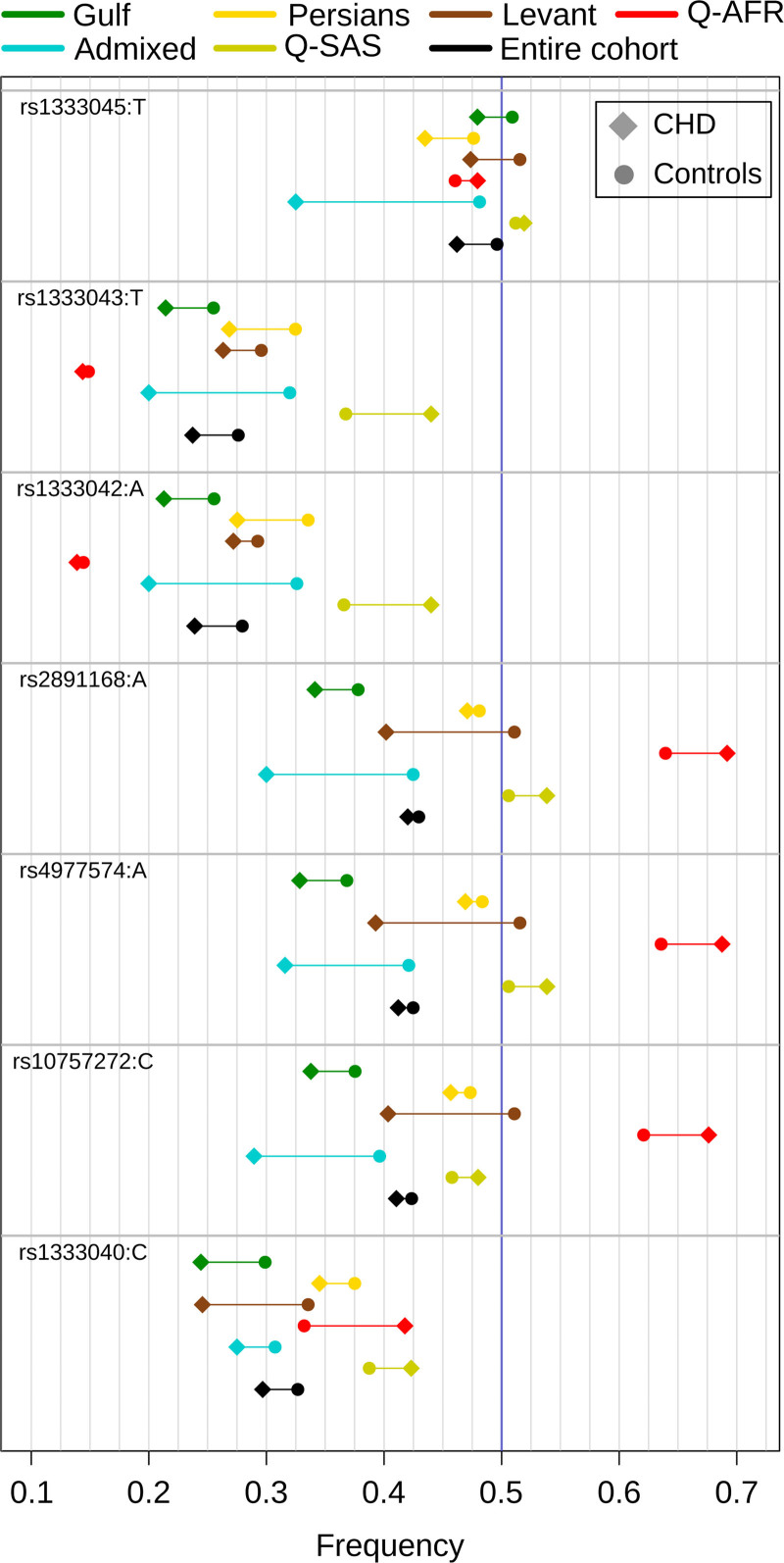
**Allele frequency heterogeneity of the 9p21 locus across ancestries for the 7 single nucleotide variant (SNVs) used for haplotype analysis.** The frequencies are for the allele shown beside the SNV rs name in each ancestry group. Each ancestry group is represented by a different color. A circle shape represents the allele frequency in controls whereas a diamond shape represents the allele frequency in coronary heart disease (CHD) patients. Vertical blue line is drawn at frequency=0.5.

Association results for the 15 selected SNVs at this locus are shown in Table S6 and Figure 4. Only rs1333042 (*P*=0.029) was nominally significant (Figure 4). The statistical power, computed with QUANTO,^29^ to identify SNVs with similar reported characteristics (OR=1.2 and MAF=0.4) at *α*=0.05 and 0.01 was 0.96 and 0.88, respectively. To check heterogeneity across ancestry groups in our cohort, association analysis at 9p21 was performed in each ancestry separately (Table S6). Within the 2 largest groups (Gulf and Persian), the lowest *P* was observed, *P*=0.017, in Gulf (rs1333040) and *P*=0.019 in Persians (rs1333050) (Table S6). Figure 5 and Figure S3 show the heterogeneity in allele frequencies and OR across ancestries. For instance, the allele frequency of the allele A in controls for rs4977574 was 0.368, 0.484, and 0.636 in Gulf, Persian, and Q-AFR, respectively (Figure 5). ORs were 0.92, 0.89, and 1.18, in Gulf, Persian, and Q-AFR, respectively (Figure S3). Although the effect size direction in Gulf and Persian individuals is consistent with European cohort studies, their magnitude is less (OR~0.83 in Europeans). The Q-AFR effect size direction is the opposite of European studies’ OR, but it is of similar magnitude (Figure S3). Haplotype analysis showed slightly greater significance (*P=*3.74×10^−3^) in the entire cohort for the protective haplotype, CCAAATT, encompassing 7 SNVs: rs1333040, rs10757272, rs4977574, rs2891168, rs1333042, rs1333043, and rs1333045. CCAAATT frequency in cases and controls was 0.17 and 0.213, respectively. The risk haplotype TTGGGAC was not significantly associated with CHD (Table S6). Haplotype analysis also showed a great heterogeneity of ORs and allele frequency across our cohort’s ancestries (Table S8). Adjusting for T2D did not change the results of the 15 tested SNVs (Table S8).

### Rare Variant Association Analysis

Across the selected genes, 29 high and 586 moderate impact variants were observed. Eleven variants were pathogenic/likely pathogenic (P/LP) as defined in ClinVar (*LDLR*:7, *LPL*:2, *LIPA*:1, and *APOE*:1; Table S7); 9 CHD patients and 9 controls had 1 of the 7 P/LP variants within *LDLR* (OR~5.97). Burden analysis was performed including varient effect predictor “high” variants and P/LP variants. The burden of rare variants in *LDLR*, *APOB*, *PCSK9*, and *ANGPTL4* was greater in CHD patients (CCR=5.39, 4.44, 1.81, and 5.93, respectively; Table 3). Only *LDLR* and *APOB* were statistically significant using SKAT (*P*<5.36×10^−6^ for *LDLR*; *P*<1.25×10^−4^ for *APOB*). Variant level results are shown in Figure S4 and Table S7. We focused on the highest CCR SNVs among the varient effect predictor “high” and “moderate” categories. For SNVs with a MAF of zero in controls, MAF was replaced with the lowest MAF among controls, thus computing a pseudo-CCR. Zero MAF SNVs in cases were ignored. Thirteen SNVs in *LPA*, *ANGPTL4*, *PCSK9*, *APOB*, *APOA5*, *LPL*, and *LDLR* showed a CCR>10. A missense variant in *LPA*, rs543542931, had the highest CCR, 23.7. Two SNVs were in *ANGPTL4*: rs538554190 (CCR=17.79) and chr19:8438638:C:A (CCR=11.85, ref=C, alt=A). Chr19:8438638:C:A is a stop gain variant with the allele A unobserved in gnomAD r2.1 and TOPMed Freeze 8. Nine SNVs had a CCR of ~11.85. Four of these are unobserved in gnomAD r2.1 and TOPMed Freeze 8 (chr1:55525234:T:G in *PCSK9*, chr2:21251222:G:T and chr2:21256226:C:G in *APOB*, and 8:19811636:G:T in *LPL*). The remaining SNVs were rs780910847 in *LPA*, rs760422549 in *APOA5*, rs202049029 in *LDLR*, rs11542065 in *LPL*, and rs373251374 in *APOB*. The latter SNV within *APOB* is a splice acceptor observed twice, both in CHD patients, but it is of uncertain significance in ClinVar. We also compared the prevalence of 2 rare *LPA* variants associated with lipoprotein(a) levels (rs10455872, c.3947+467T>C and rs3798220, p.Ile1891Met); rs10455872 was more frequent in CHD patients (CCR=1.14) and rs3798220 was more frequent in controls (CCR=0.3).

**Table 3.**
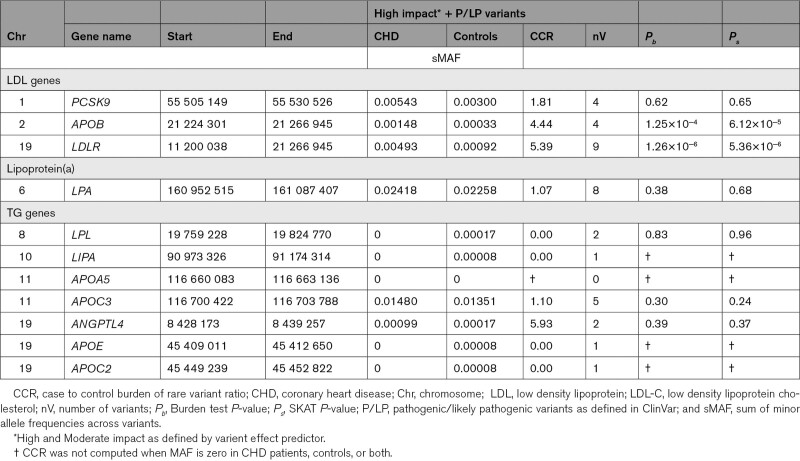
Burden of Rare Variants for LDL-C, TG, and Lipoprotein(a) Genes in both CHD Patients and Controls

### Plausible Genes With Biological Links to CHD and Expression Quantitative Trait Loci Evidence

Three of the most significant loci (*P*<10^−5^) mapped to previously reported CHD genes: *ABCG8*, *SLC22A4/5*, and *MIR548A3*. Other SNVs were in the vicinity of genes that are plausibly linked to CHD (*CORO7-PAM1/CORO7/VASN*, *PDE4D*, *RBM47*, *TNS3*, *HS3ST1*, and *HS3ST3B1*). Significant expression quantitative trait loci evidence with the most significant SNVs was also observed for plausible CHD genes: *SNX17*, which is known to be associated with triglycerides,^30^ was associated with rs1260330 (*P*=7.03×10^−75^) in skeletal muscles; *CORO7* was associated with rs917306 in whole blood (*P*=1.91×10^−4^); *MARK3* was associated with rs35696698 in the left ventricle of the heart (*P*=6.67×10^−3^) and was previously reported to be associated with CHD.^31^ Summary of these findings are shown in Table S8, and description of gene functions can be found in the Supplemental Material. Expression quantitative trait loci results can be found in Table S9.

## Discussion

We performed WGS of a CHD case-control cohort to characterize the genetic architecture of CHD in the ME, one of the most underrepresented populations in GWAS.^32^ We replicated several known CHD loci and identified potential novel loci. PRSs derived from European ancestry cohorts performed well, suggesting their potential use for risk stratification in ME cohorts.

Enthusiasm for using PRSs in the clinical setting is dampened by concerns about their portability across diverse ancestry groups.^33^ PRSs derived from European ancestry cohorts performed surprisingly well in the Qatari cohort. Our results, along with other reports, suggest that genome-wide PRSs may port well to individuals of South Asian,^16^ Latino,^13^ and ME ancestries. Excepting lassosum, the PRSs we evaluated provided good discriminative power between cases and controls. EnsemblePRS, which combined multiple PRSs, outperformed other PRSs, possibly by capturing orthogonal information from each PRS. The use of WGS data likely improved the performance, by reducing the “noise” of imputed data. Low pass WGS data has been shown to outperform genotype data when developing PRSs.^15^ The robust performance of PRSs in our cohort suggests an overlap of genetic susceptibility across ancestries. Additional CHD GWASs in the ME and Gulf region may lead to new discovery of variants and development of trans-ancestry PRSs that could outperform single ancestry PRSs.

Our findings have potentially significant clinical implications. CHD is rampant in the region, and rates of CHD in younger individuals are rising.^23^ There is an imperative to detect high-risk individuals early on so that preventive measures can be implemented. The use of PRSs with conventional risk factors may help identify those at highest risk.^34^ Our results showed that 10% of the cohort with the highest PRS had an odds ratio >3.45 for CHD, which is equivalent to the risk from a monogenic disorder such as familial hypercholesterolemia. Lifestyle measures and statin therapy could be instituted to reduce the risk in such individuals.^35^

Our study replicated 33 of 400 known CHD loci and identified 6 suggestive new loci, including genes with biological functions related to CHD (eg, *CORO7/CORO7-PAM16/VASN*, *PDE4D*, and *RBM47*). Interestingly, the well-established susceptibility locus 9p21 (*CDKN2A/2B*) was not replicated. Haplotype analysis revealed 1 protective haplotype to be associated at a nominally significant level. In a recent study,^36^ the 9p21 locus did not reach genome-wide significance among Black individuals, nor among Hispanic individuals, even after 2-stage meta-analysis involving >27 000 and >12 100 CHD cases, respectively. The heterogeneity across our cohort ancestries observed for the 9p21 locus in term of effect size and allele frequencies coupled with the small sample size of some of the ancestry groups highlights the need of a larger cohort with similar characteristics. Nonetheless, ORs within the largest groups in our cohort were lower than what was reported in European studies. To the best of our knowledge, no GWAS study for CHD in ME populations showed robust significant association with 9p21. For example, a study in a Lebanese population reported a *P*=0.01 with CHD.^37^

Our study also highlights the utility of WGS for stratifying the risk of CHD. WGS can not only identify actionable monogenic variants, but also provide polygenic risk estimates and identify variants with pharmacogenetic implications. We were able to compute a PRS for CHD as well as identify a greater burden of rare variants in genes known to underlie monogenic disorders of lipid metabolism, leading to increased CHD risk. We found a greater burden of *LPA* risk alleles in cases versus controls, supporting the causal role of lipoprotein(a) for CHD in a ME cohort. As sequencing costs decrease, one can foresee the use of WGS in early adulthood to identify high-risk individuals for both common and rare disorders for which preventive options are available, thereby reducing the morbidity and mortality.

Eighteen individuals (9 CHD patients and 9 controls) had P/LP variants in *LDLR*. *LDLR*, *APOB*, *PCSK9*, and *ANGPTL4* showed the highest burden increase in CHD. Some high-impact variants were more frequent in our cohort suggesting a founder effect, such as rs76353203 within *APOC3* (MAF=~0.014 in our cohort versus ~0.002 in gnomAD r2.1). There are conflicting interpretations of pathogenicity in ClinVar and debate whether this variant is protective against CHD. Our results only showed a small increase in cases (CCR=1.11).

Although we limited our study to Qataris, population structure analysis revealed significant population strata within the cohort, as previously reported,^38^ with the major groups being Gulf, Persian, Levant, African, South Asian, and Admixed. These results highlight population stratification even in cohorts from a discrete geographical region and the need to correct for such stratification. The lack of a replication stage with a dataset that matches our discovery cohort limited our ability to replicate our findings and develop an ancestry-specific PRS. A challenge is that native Qataris (population ~2 600 000), only comprise 12% of Qatar’s total population. Although we used GWAS summary statistics to replicate our findings and conducted expression quantitative trait loci analysis using GTEx V8.0 data to further validate/prioritize genes, there is a need for generating additional omics datasets in underrepresented populations.

In conclusion, in the first WGS study of CHD from the ME region, we replicated several known CHD loci, but not the major common susceptibility locus for CHD, 9p21. Several of our most significant SNVs were replicated using summary statistics from available European ancestry GWAS. PRSs derived from European ancestry cohorts performed well, suggesting these could be used in the clinical setting while ancestry-specific PRSs are developed. Additionally, we highlighted the differential burden of rare variants within genes involved in lipoprotein metabolism between cases and controls. Finally, our results suggested new plausible susceptibility loci for CHD that need to be replicated in further studies.

## Article Information

### Acknowledgments

The authors gratefully acknowledge individuals who provided biological samples and data for QCBio and QBB. The authors would like to thank the Qatar Biobank and Qatar Genome Program for their support. The authors also thank Yusef Kunji for valuable discussions and comments on biochemical pathways and gene function and Fayaz Mir for the help with sample acquisition in QCBio.

I.J.K., J.A.S., A.E.M. conceived the study. I.J.K., J.A.S., and M.S. designed the analysis plan. J.A.S. and A.E.M. collected CHD patients. Q.G.P./Q.B.B. provided the control cohort. Q.G.P. supported with the sequencing of CHD patients. M.S., K.K., and E.U. had access to the genomics data. M.S., K.K., and E.U. performed the statistical analysis. M.S., K.K., and E.U. generated the figures and tables. All authors contributed to result interpretation. M.S. drafted the article, and all co-authors revised it and approved it. J.A.S. coordinated the study.

### Sources of Funding

This study was supported in part by the Qatar National Research Fund (NPRP: 5-1024-3-225 with MRC: MRC-01-17-005) and the Qatar Genome Program. I.J.K. was additionally funded by National Institutes of Health grants HG011710, HG06379, and HL137010. Open Access funding was provided by the Qatar National Library. The other authors report no conflicts.

### Disclosures

None.

### Supplemental Material

Expanded Methods

Tables S1–S10 [S2, S3, S5, S7, S9 separate excel file]

Figures S1–S4

Supplemental Methods and Results

References^39–73^

## Supplementary Material
