# Supplementary material for: Validation of Polygenic Risk Scores for Coronary Heart Disease in a Middle Eastern Cohort Using Whole Genome Sequencing
Source: Circ Genom Precis Med. 2022 Oct 12;15(6):e003712. doi: 10.1161/CIRCGEN.122.003712 (PMC9770120; doi:10.1161/CIRCGEN.122.003712)
Supplement: Supplementary file 1 [file hcg-15-e003712-s001.pdf]

SUPPLEMENTAL MATERIAL

Expanded Methods

Whole genome sequencing data and quality control

WGS was performed at the Sidra Clinical Genomics Laboratory at 30x coverage depth. Raw sequencing reads were aligned to human reference build 37 (hs37d5) and were processed using the GATK best-practices pipeline to obtain genotype calls in the Variant Call Format. Additional details can be found in elsewhere,[39,40] and in the below **Supplemental Methods**.

Low-quality genotypes were converted to missing. SNVs were removed based on poor sequencing quality, deviation from Hardy-Weinberg Equilibrium, excess of missing genotypes, and excess of heterozygosity. Individuals were removed based on excess of missing genotypes, excess of heterozygosity, relatedness, and sex ambiguity (see details in the below **Supplemental Methods**). After QC steps, 1,014 CHD cases and 6,009 controls remained. For association analysis, autosomal biallelic SNVs that are not in low complexity regions as defined by Li, H.[41] and with minor allele frequency (MAF) >0.01 were tested (8,278,388 SNVs).

Statistical analysis

Relatedness and population structure inference

Kinship coefficients were calculated using PLINK v1.09[42] to remove duplicated or highly related subjects. Genetic relationship matrix (GRM) (incorporated in association analyses) was constructed using GCTA.[43] PC-AiR[44] was run on a set of independent SNVs to assess population stratification while accounting for relatedness inferred by GCTA. The first 20 principal components (PCs) were included as

covariates in the association analysis. To identify ancestry groups in our cohorts, we ran another PC-AiR analysis, combining our data and the 1000 Genomes Project (1KG) data of the five major ancestries.

Polygenic risk scores

We followed the recently recommended framework for reporting polygenic risk studies.[26] In our cohort, we assessed the performance of 5 PRSs that were developed with various methods and using various types of data and ancestries. PRS training and evaluation in the original studies were based on multi-ancestry, South Asian, East Asian, and European cohorts. The methods for developing the PRS were pruning and thresholding (PGS000337 and PGS000749),[28,45] lassosum (PGS000116),[34] LDpred (PGS000296),[16] and metaGRS (PGS000018).[12] PRSs were downloaded from the polygenic risk score catalog (https://www.pgscatalog.org). Characteristics of PRSs, i.e., number of SNVs, training and evaluation ancestry, and performance in original studies, are described in **Supplemental Table V**. PRSs were derived using different statistical techniques and ancestries, capturing orthogonal information. We calculated an EnsemblePRS by summing PRSs after centering the mean and standardizing each PRS. PRSs were computed using the PLINK 1.09 '--score' command, then mean-centered and scaled with respect to controls. A logistic regression model was used to determine association between CHD and PRS, adjusting for age, sex, BMI, and 20 PCs. OR per 1 SD increase (OR_{1sd}) of each PRS was calculated. Area under the receiver-operator curve (AUC) was calculated using R package pROC (https://cran.r-project.org/web/packages/pROC/pROC.pdf) for a model that only contained the PRS and PCs because of ascertainment bias with respect to age and sex. The PRS was binned into 10 deciles and the proportion of CHD patients within each decile group was compared to either all lower decile groups combined (OR_{vsAll}), or the lowest decile group ($OR_{vsLowest}$), using a logistic regression model that included all covariates. To test robustness of PRS results, a subset of controls was selected based on age and sex to match cases. Several models including different combinations of covariates were performed on this age/sex matched data, and on the entire cohort.

Association analysis for common variants

Main association analysis was conducted using GMMAT's generalized linear mixed model and its score test[46] to account for relatedness and to adjust for age, sex, body mass index (BMI), and

the first 20 PCs. A Wald test was performed on the most significant SNVs to calculate odds ratios and confidence intervals. Other models with various combinations of covariates were also performed. The standalone version of LocusZoom[47] was used to generate regional plots that show the linkage disequilibrium between SNVs (extracted from our data) and association results. For the most significant SNVs, expression quantitative trait locus (eQTL) analysis was performed using GTEx V8.0 data in several relevant tissues (see below **Supplemental Methods** for more details). Evidence of association between SNVs and expression of neighboring genes within 1 MB was considered.

Haplotype analysis for the 9p21 locus

Fifteen SNVs at the 9p21 locus, reported to be associated with CHD in European ancestry cohorts, were considered in this analysis; these are located on chromosome 9 between 22,040,765 and 22,125,913 and include: rs1333037, rs1333036, rs1333039, rs1333040, rs10757272, rs4977574, rs2891168, rs1333042, rs1333043, rs1333045, rs1333046, rs1333047, rs1333048, rs1333049, and rs1333050. Haplotypes were obtained using a sliding window of seven SNVs, using PLINK v1.07.[48] The association test for each haplotype (presence or absence) was performed using GMMAT's Wald test, including the same covariates and accounting for relatedness.

Genomic loci associated with CHD

A list of previously identified CHD loci from all studied ancestry groups was extracted from the GWAS catalog and then supplemented with results from recent relevant publications.[10,49-51] The final set contained 936 unique SNVs **(Supplemental Table VI)**, spanning 400 independent loci based on LD-pruning (PLINK '--indep 50 5 1.05') conducted on the 1000 Genomes Project (1KG) European ancestry data.[25]

Association analysis for rare variants

We limited rare variant analysis to lipoprotein metabolism genes: *APOB*, *LDLR*, and *PCSK9* in the LDL-cholesterol pathway, *APOA5*, *APOC2*, *LPL*, *LIPA*, *APOC3*, *APOE*, and *ANGPTL4* in the triglyceride pathway, and lipoprotein(a) *LPA*. Bi- and multi-allelic variants within these genes were annotated using the Ensembl Variant Effect Predictor (VEP; https://grch37.ensembl.org/Tools/VEP) to predict the impact of variants ('high' and 'moderate'). Additionally, we assessed the prevalence of pathogenic/likely

pathogenic (P/LP) variants in these genes (ClinVar; https://www.ncbi.nlm.nih.gov/clinvar/; May 5, 2021) in cases and controls. We selected P/LP, 'high', and 'moderate' variants with MAF<0.05, GATK filter value of 'PASS', 'ExcessHet'<54, 'InbreedingCoeff'> -0.2, and missing genotype rate <0.05. MAF was compared between cases and controls at a variant-level, and at a gene-level by aggregating variants within genes. The burden of rare variants was calculated as the sum of the MAF of variants within each gene. The case to control ratio (CCR) of the rare variant burden was calculated as $\mathrm{CCR} = \frac{\sum \mathrm{MAF}_{i,\mathrm{CHD}}}{\sum \mathrm{MAF}_{i,\mathrm{Controls}}}$ where i represents the variants. SKAT and burden test were performed using the SMMAT R package.[46]

Supplemental Table Legends

Supplemental Table I: PRS results for various models with different combinations of covariates

Supplemental Table II: The details about the nine GWAS summary statistics used for meta-analysis in the replication stage

Supplemental Table III: Meta-analysis of the most significant SNVs in public GWAS summary statistics described in **Supplementary Table II** where meta-analysis $P < 10^{-3}$

Supplemental Table IV: Summary of findings, and biological functions of the identified plausible genes

Supplemental Table V: List of PRSs that were evaluated in our study and their characteristics

11 Non-referenced figures and 1 non-referenced table (DeLong's statistical test for AUC differences) are in the **Supplemental Methods and Results** section below.

Supplemental Table I: PRS results for various models with different combinations of covariates

		Entire cohort							Age and Sex matched						
**PRS**	**Covariates**	**OR**	**OR_L**	**OR_U**	**AUC**	**AUC_L**	**AUC_U**	***P***	**OR**	**OR_L**	**OR_U**	**AUC**	**AUC_L**	**AUC_U**	***P***
PGS000337	None	1.759	1.644	1.881	0.656	0.638	0.674	1.63E-60	1.708	1.602	1.821	0.651	0.634	0.668	3.04E-60
PGS000337	Age+Sex	1.889	1.737	2.053	0.901	0.892	0.910	2.72E-50	1.769	1.656	1.889	0.680	0.664	0.696	7.08E-65
PGS000337	Age+Sex+BMI	1.883	1.732	2.048	0.901	0.892	0.910	8.18E-50	1.770	1.658	1.891	0.680	0.664	0.697	5.64E-65
PGS000337	Age+Sex+BMI+20 PCs	1.812	1.662	1.976	0.905	0.896	0.914	3.07E-41	1.735	1.619	1.858	0.703	0.687	0.719	1.65E-55
PGS000337	20 PCs	1.728	1.612	1.852	0.667	0.649	0.685	1.18E-53	1.679	1.570	1.795	0.677	0.660	0.693	5.74E-52
PGS000296	None	1.620	1.527	1.718	0.683	0.664	0.701	1.17E-57	1.773	1.655	1.901	0.685	0.668	0.701	3.68E-59
PGS000296	Age+Sex	1.593	1.486	1.708	0.901	0.892	0.910	2.30E-39	1.810	1.686	1.942	0.701	0.685	0.717	5.90E-61
PGS000296	Age+Sex+BMI	1.591	1.485	1.706	0.901	0.892	0.910	1.99E-39	1.812	1.689	1.945	0.701	0.685	0.717	4.19E-61
PGS000296	Age+Sex+BMI+20 PCs	1.530	1.424	1.643	0.904	0.895	0.913	3.47E-31	1.764	1.635	1.904	0.714	0.698	0.729	1.18E-48
PGS000296	20 PCs	1.597	1.501	1.700	0.683	0.665	0.701	2.90E-49	1.744	1.619	1.879	0.695	0.679	0.711	2.83E-48
PGS000018	None	1.654	1.555	1.759	0.684	0.666	0.702	1.29E-57	1.804	1.681	1.937	0.681	0.665	0.697	4.09E-60
PGS000018	Age+Sex	1.613	1.503	1.732	0.901	0.892	0.910	1.20E-39	1.848	1.719	1.987	0.700	0.684	0.716	3.56E-62
PGS000018	Age+Sex+BMI	1.611	1.501	1.730	0.901	0.892	0.910	1.11E-39	1.852	1.722	1.991	0.701	0.685	0.717	2.10E-62
PGS000018	Age+Sex+BMI+20 PCs	1.539	1.431	1.656	0.904	0.895	0.913	5.07E-31	1.786	1.652	1.930	0.712	0.697	0.728	2.16E-48
PGS000018	20 PCs	1.628	1.525	1.737	0.686	0.667	0.704	8.92E-49	1.760	1.631	1.900	0.691	0.675	0.707	7.66E-48
PGS000749	None	1.587	1.486	1.695	0.631	0.613	0.649	2.62E-43	1.604	1.506	1.708	0.636	0.619	0.653	3.02E-49
PGS000749	Age+Sex	1.696	1.564	1.838	0.896	0.887	0.905	1.09E-37	1.644	1.542	1.753	0.661	0.644	0.678	2.14E-52
PGS000749	Age+Sex+BMI	1.691	1.560	1.833	0.896	0.887	0.905	2.63E-37	1.647	1.545	1.756	0.662	0.645	0.678	1.35E-52
PGS000749	Age+Sex+BMI+20 PCs	1.655	1.506	1.819	0.900	0.891	0.909	1.35E-25	1.658	1.537	1.788	0.685	0.668	0.701	3.10E-39
PGS000749	20 PCs	1.636	1.515	1.767	0.645	0.627	0.663	6.82E-36	1.627	1.511	1.752	0.661	0.644	0.678	4.98E-38
EnsemblePRS	None	1.814	1.702	1.933	0.694	0.676	0.712	7.98E-75	1.896	1.773	2.027	0.694	0.678	0.710	8.27E-78
EnsemblePRS	Age+Sex	1.846	1.710	1.993	0.906	0.897	0.915	1.45E-55	1.962	1.832	2.103	0.715	0.699	0.730	9.43E-82
EnsemblePRS	Age+Sex+BMI	1.842	1.707	1.988	0.906	0.897	0.915	2.43E-55	1.967	1.835	2.107	0.716	0.700	0.731	5.04E-82
EnsemblePRS	Age+Sex+BMI+20 PCs	1.800	1.657	1.956	0.909	0.900	0.918	5.89E-44	2.012	1.860	2.176	0.729	0.714	0.744	3.58E-68
EnsemblePRS	20 PCs	1.858	1.731	1.995	0.702	0.684	0.720	2.94E-65	1.954	1.810	2.109	0.708	0.692	0.724	4.89E-66

OR_L: lower confidence interval boundary of OR; OR_U: upper confidence interval boundary of OR; AUC_L: lower confidence interval boundary of AUC; AUC_U: upper confidence interval boundary of AUC;

Supplemental Table II: The details about the nine GWAS summary statistics used for meta-analysis in the replication stage

Study	Cases	Controls	Ethnicity	Publication Year	Cohort	Summary Statistics File names
Schunkert H, König IR, Kathiresan S, Reilly MP, Assimes TL, Holm H et al. Large-scale association analysis identifies 13 new susceptibility loci for coronary artery disease. Nat Genet. 2011 43: 333-338	22233	64762	EUR	2011	GWAS+HapMap2 imputation	cardiogram_gwas_results*
Peden JF, Hopewell JC, Saleheen D, Chambers JC, Hager J, Soranzo N, Collins R, Danesh J, Elliott P, Farrall M, Stirrups K, Zhang W, Hamsten A, Parish S, Lathrop M, Watkins H (Chair), Clarke R, Deloukas P, Kooner J). A genome-wide association study in Europeans and South Asians identifies five new loci for coronary artery disease. Nat Genet. 2011 43: 339-344	15420	15062	EUR+SAS	2011	GWAS Illumina Whole-genome Beachips, ~500K SNPs without imputation; rare and common variants	c4d_cad_discovery_metaanalysis*
CARDIoGRAMplusC4D Consortium, Deloukas P, Kanoni S, Willenborg C, Farrall M, Assimes TL, Thompson JR, et al. Large-scale association analysis identifies new risk loci for coronary artery disease. Nat Genet 2013 45:25-33	63746	130681	EUR+SAS	2013	GWAS metabochip + GWAS imputed with HapMap	cardiogramplusc4d_data*
CARDIoGRAMplusC4D Consortium, M Nikpey, A Goel, H Won, LM Hall C. Willenborg, S Kanoni, D Saleheen et al. A comprehensive 1000 Genomes–based genome-wide association meta-analysis of coronary artery disease. Nat Genet 2015 47:1121-1130	60801	123504	EUR	2015	GWAS imputed with 1KG	cad.additive.Oct2015.pub*
Myocardial Infarction Genetics and CARDIoGRAM Exome Consortia Investigators, Stitziel NO, Stirrups KE, Masca NG, Erdmann J, et al. Coding Variation in ANGPTL4, LPL, and SVEP1 and the Risk of Coronary Disease/. N Engl J Med. 2016 ||| Webb TR, Erdmann J, Stirrups KE, Stitziel NO, Masca NG, et al. Systematic Evaluation of Pleiotropy Identifies 6 Further Loci Associated With Coronary Artery Disease. J Am Coll Cardiol. 2017 Feb	42335	78240	EUR	2017	Exome-chip	MICAD.EUR.ExA.Consortium.PublicRelease.310517*
CP Nelson, A Goel, AS Butterworth, S Kanoni, TR Webb, et al. Association analyses based on false discovery rate implicate new loci for coronary artery disease. Nat Genet 2017 Jul 17 49(9): 1385-1391. doi: 10.1038/ng.3913	10801	137914	EUR	2017	GWAS + imputation 1KG: SOFT CAD cases=10801, HARD CAD=6482	UKBB.GWAS1KG.EXOME.CAD.SOFT.META.PublicRelease.300517.txt*
genome-wide analyses identify distinct and shared genetic risk loci for coronary artery disease. Nat Genet. 2020;52:1169-1177	25892	142336	Japanese	2020	GWAS + imputation	https://humandbs.biosciencedbc.jp/en/hum0014-v26
FinnGen	7661	85760	EUR Finnish		GWAS + imputation	http://r2.finngen.fi/pheno/I9_CORATHER
SAIGE UKBB	20023	377103	EUR White British		GWAS + imputation	http://pheweb.sph.umich.edu/SAIGE-UKB/pheno/411.4

*http://www.cardiogramplusc4d.org/data-downloads/

Supplemental Table III: Meta-analysis of the most significant SNVs in public GWAS summary statistics described in **Supplemental Table II** where meta-analysis $P < 10^{-3}$

Chr	rsName	BP37	Nearest Gene	Dst	Minor	Major	MAF	OR [CI]	*P*	MAF cases	MAF controls	Meta-analysis *P*	K	Known	CHD genes
1	rs7528419	109,817,192	*CELSR2*	0	G	A	0.167	0.7 [0.58,0.83]	6.29E-05	0.139	0.171	$<10^{-300}$	7	yes	*SORT1,CELSR2*
2	rs114309202	44,087,612	*ABCG8*	0	G	A	0.084	1.65 [1.33,2.04]	3.77E-06	0.109	0.079	3.41E-06	4	yes	*DYNC2LI1,PLEKHH2,ABCG5,ABCG8*
11	rs142264789	5,772,160	*TRIM5*	0	A	G	0.020	0.3 [0.16,0.55]	8.42E-05	0.009	0.022	4.58E-05	4	yes	*TRIM5,TRIM22*
4	rs17033109	156,446,231	*RP13-487K5.1*	8	G	A	0.155	0.69 [0.58,0.83]	5.22E-05	0.1289	0.1595	1.49E-04	6	yes	*intergenic,GUCY1A3,GUCY1A1,MAP9*
19	rs62119261	45,122,043	*CEACAM22P*	0	C	A	0.063	1.66 [1.29,2.14]	9.12E-05	0.069	0.062	1.49E-04	4	yes	*APOE,APOC1,TOMM40*
14	rs35696698	103,230,573	*RP11-661D19.3*	-6	T	G	0.191	0.71 [0.6,0.83]	3.55E-05	0.161	0.196	2.12E-04	4	no	-

Chr: Chromosome; BP37: GRCh37 positions; Nearest gene: nearest gene to the lead SNV or the gene that contains the SNV; Dst: Distance (kb) from neartest gene (0 if within the gene); Minor: Minor Allele; Major: Major allele; MAF: Minor Allele Frequency; OR [CI]: Minor Allele Odds ratio and 95% Confidence Interval; Known: Yes of the SNV was within 300KB of a known CHD loci, listed in Supplementary Table 1. *P* : P-value of the association score test by GMMAT; Meta-analysis *P* : P-value from inverse-variance meta-analysis; K: Number of studies in the meta-analysis; Known: "yes" if the SNV exists in Supplementary Table 1 and "no" otherwise; CHD genes: reported CHD genes.

Supplemental Table IV: Summary of findings, and biological functions of the identified plausible genes

Chr	rsName	Rep	Gene Name	eQTL SNV	eQTL *P*	GTEx tissue	CHD risk factors	Biological function relevant to CHD
2	rs1260330	-	*SNX17*	rs1260330	7.03E-75	Muscle Skeletal	Total cholesterol levels, LDL-C, Triglyceride levels	*SNX17* required for maintenance of normal cell surface levels of APP and LRP1
4	rs7690530	-	*RBM47*	-			Systolic and diastolic blood pressure, Hypertension, Total Cholesterol Measurement, LDL-C, ApoB Measurement, Carotid Artery Intima Media Thickness	*RBM47* is a paralog of *A1CF* (APOBEC1 Complementation Factor) and is also a cofactor for mRNA editing of ApoB by APOBEC (APOBEC1) to produce the shortened ApoB-48.
7	rs35614549	+	*GAPDHP68*	-			Transforming growth factor beta measurement, FEV change measurement, idiopathic dilated cardiomyopathy, BMI-adjusted waist-hip ratio	
13	rs513479	-	*PROZ*	rs513479	1.57E-09	Artery Tibial		*PROZ* appears to assist hemostasis by binding thrombin and promoting its association with phospholipid vesicles. It also inhibits activity of the coagulation protease factor Xa in the presence of SERPINA10, calcium, and phospholipids
16	rs917306	-	*CORO7/CORO7-PAM16/VASN*	rs917306	1.91E-04	Whole Blood	Systolic Blood Pressure, Waist-Hip Ratio	VASN modulates arterial response to injury in vivo and has lower arterial wall expression with age. CORO7 repression prevents clearance of LDLR-A18-GFP (and hence likely untagged LDLR) from the Golgi Apparatus and mutations in *CORO7-PAM* reduce the rate of LDLR clearance.
4/17	rs114906338/rs12950395	-	*HS3ST1/HS3ST3B1*	-				*HS3ST1* is the rate limiting 3-O sulfotransferase in the synthesis of anticoagulant heparan sulfate and completes the antithrombin pentasacharide binding site. *HS3ST3B1 is also a* 3-O sulfotransferase at the same step as HS3ST1, but instead results in the synthesis of non-anticoagulant heparan sulfate.
1	rs7528419	+	*CELSR2*	rs7528419	2.23E-76	Muscle Skeletal		Genes previously discovered in GWAS
2	rs114309202	+	*ABCG8*	-				
5	rs75413826	-	*P4HA2/SLC22A4*	-				
11	rs142264789	+	*TRIM5*	-				
14	rs35696698	+	*MARK3*	rs35696698	6.67E-03	Heart Left Ventricle		
19	rs62119261	+	*APOE*	rs62119261	3.30E-02	Artery Tibial		

Rep: "Y" if SNV was replicated with meta-analysis $P < 10^{-3}$. eQTL: SNV that was associated with the corresponding gene expression (P<0.05) in GTEx without FDR adjustement; eQTL *P*: eQTL *P* between the SNV and the gene expression; GTEx tissue: tissue where evidence of eQTL was observed; CHD risk factors: a subset of relevant CHD risk factors that were associated with the corresponding gene as shown in GWAS catalog; Biological function relevant to CHD: biological function of the corresponding gene that may be linked to CHD. Detailed gene functions can be found in Supplementary Material. For genes that were previously reported to be associated with CHD, CHD risk factors and biological functions were not reported. List of SNVs were split in two parts: the potentially novel genes and genes previously discovered in GWAS of CHD. The SNVs were ordered by chromosome and position.

Supplemental Table V: List of PRSs that were evaluated in our study and their characteristics

PGS ID*	Nvar	O	Reference	Performance	Training#	Evaluation#
PGS000116	40,079	39,194	Elliott J et al. JAMA (2020)	C-index = 0.61	Multi-ancestry	Multi-ancestry
PGS000337	75,028	74,570	Koyama S et al. Nat Genet (2020)	AUC=0.67; OR=1.84	Multi-ancestry	East Asian
PGS000749	1,056,021	1,028,613	Gola D et al. Circ Genom Precis Med (2020)	AUC=0.59 - 0.61	European	European
PGS000018	1,745,179	1,669,697	Inouye M et al. J Am Coll Cardiol (2018)	C-index = 0.62	Multi-ancestry	Multi-ancestry
PGS000296	6,630,150	6,146,138	Wang M et al. J Am Coll Cardiol (2020)	^AUC = 0.79 - 0.80; ^OR = 1.38 - 1.58	South Asian	South Asian

^: This is the name of the PRS we use in the manuscript; *: PGS ID in PGS catalog (https://www.pgscatalog.org/); Nvar: number of variants included in the PRS; O: number of overlapping variants in our study. ^Model includes sex, age, and 5 PCs as covariates; #: Information extracted from the PGS catalog.

Supplemental Table VI: Association results of the most significant SNVs (P < 10-4) [see separate excel]

Supplemental Table VII: Known CHD SNVs extracted from the GWAS catalog and supplemented by recent study results [see separate excel]

Supplemental Table VIII: Single marker and haplotype association results for 15 SNPs in the 9p21 region that have been strongly associated with CHD in prior GWASs [see separate excel]

Supplemental Table IX: Rare variants within LDL-C pathway, TG pathway, and Lipoprotein(a) genes in both CHD patients and controls for high and moderate impact variants as annotated by VEP and P/LP annotated in ClinVar [see separate excel]

Supplemental Table X: GTEx eQTL results for the most significant SNVs (P < 10-4) [see separate excel]

Supplemental Figure I: PRS decile plot for $OR_{vsLowest}$

Decile plots showing $OR_{vsLowest}$ (see methods). Bars represent the 95% $OR_{vsLowest}$ confidence interval. The numbers below the bars are the $OR_{vsLowest}$ values.

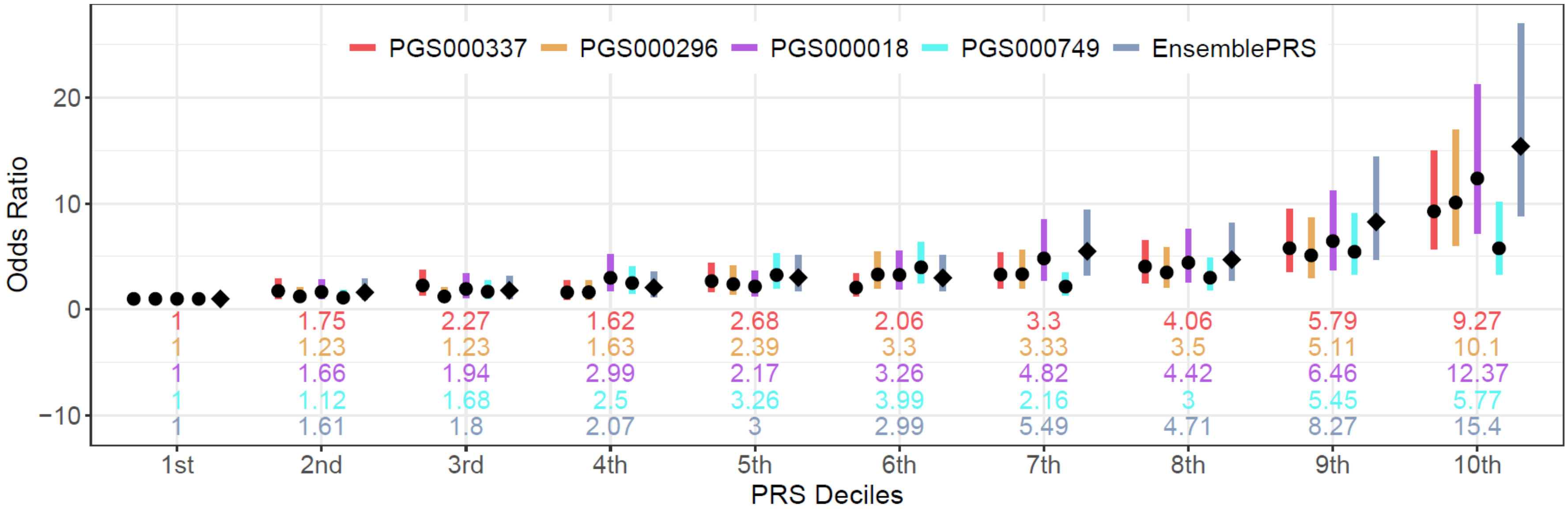

Supplemental Figure II: Manhattan and QQ plots

QQ plots are shown for different MAF intervals: black for MAF ≤ 0.05, red for 0.05 < MAF ≤ 0.2, green for 0.2 < MAF ≤ 0.5, and blue for MAF ≤ 0.5. λ is the genomic inflation factor. It is calculated for different quantiles (0.5, 0.1, 0.01, and 0.001). MAF = minor allele frequency.

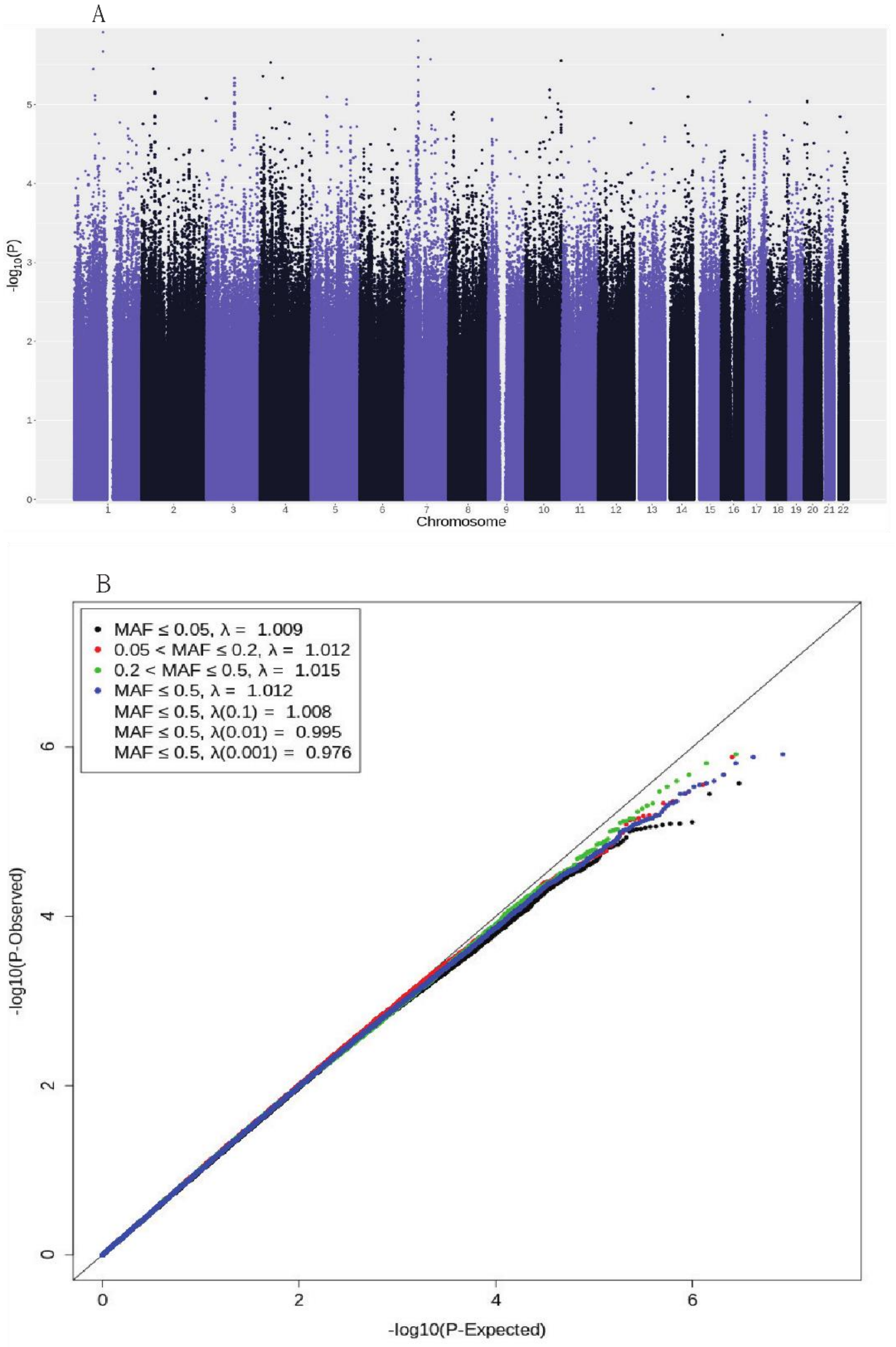

Supplemental Figure III:

Heterogeneity of ORs at the 9p21 locus across ancestries for the 7 SNVs used for haplotype analysis

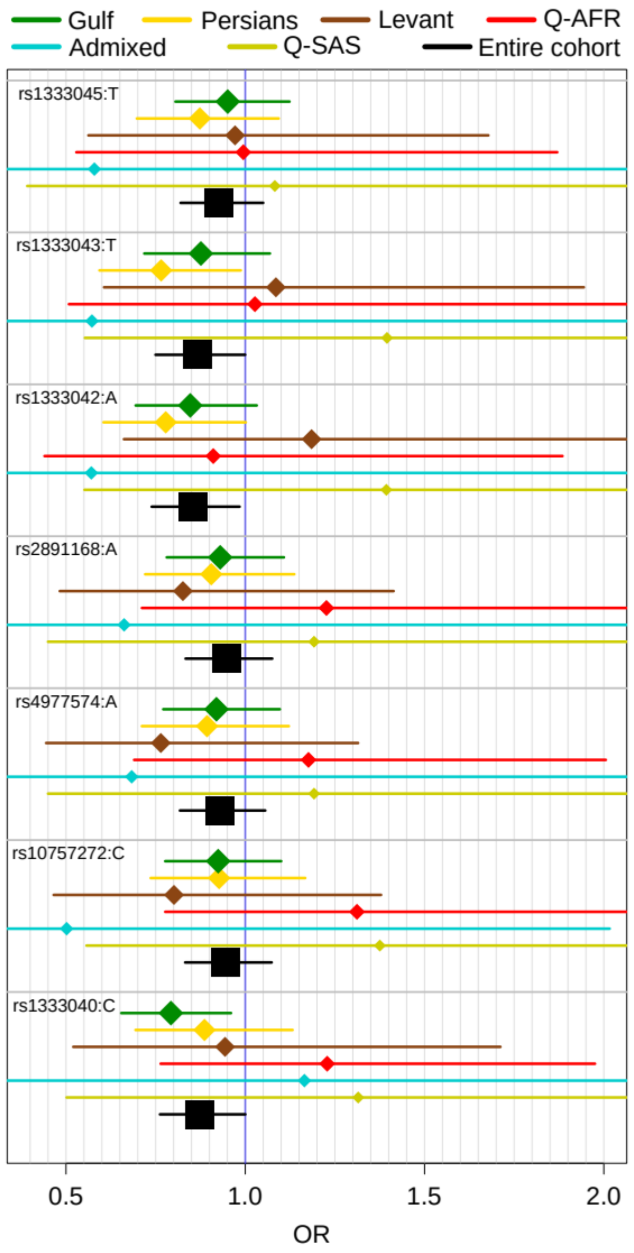

Supplemental Figure IV: Rare variant association analysis in lipid genes

CCR of the 'high' and 'moderate' impact variants annotated by VEP, and pathogenic/likely pathogenic defined by ClinVar. The vertical line is CCR=1. Filled circles are the risk variants. Empty circles are the protective variants. Variant names are shown only for SNVs with CCR>8. CCR=case to control ratio; VEP=variant effect predictor.

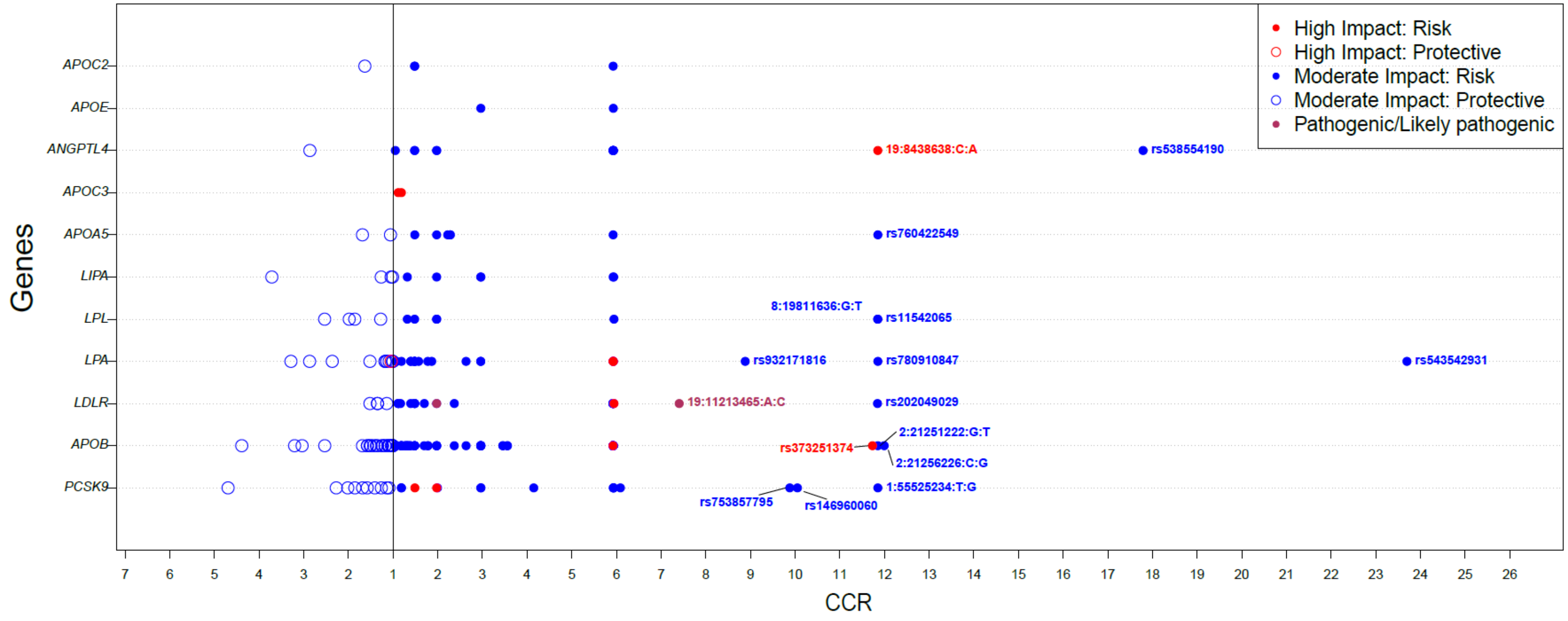

Supplemental Methods and Results

Whole genome sequencing data

Whole genome sequencing for both cases and controls was performed at Sidra medicine with 30x depth of coverage. DNA samples were extracted from peripheral blood using the Qiagen MIDI kit protocol's recommendations (Qiagen, Germany) for the automated QIASymphony SP instrument. The Caliper Labchip GXII (Perkin Elmer, USA) was used for assessment of DNA integrity. The Quant-iT dsDNA Assay (Invitrogen, USA) was used for quantification on the FlexStation 3 (Molecular Devices, USA). The Illumina TruSeq DNA Nano kit was used with 150 ng of DNA to prepare whole genome libraries and sequencing was performed on HiSeq X Ten (illumina, USA). WGS Sequencing and library preparation were performed by the Sidra Clinical Genomics Laboratory Sequencing Facility. Quality control (QC) was evaluated using FastQC (v0.11.2) (https://www.bioinformatics.babraham.ac.uk/projects/fastqc/). Quality control on mapped reads was performed using Picard (v1.117) [CollectWgsMetrics] (https://gatk.broadinstitute.org/hc/en-us). Raw sequencing reads were aligned to human reference build 37 (hs37d5) using BWA-MEM (v0.7.12-r1039). Aligned reads were further processed using the GATK best-practices pipeline (https://gatk.broadinstitute.org/hc/en-us/articles/360035535932-Germline-short-variant-discovery-SNPs-Indels-) to obtain the genotype calls in Variant Call Format (VCF). The bioinformatics pipeline used to generate VCF files is described elsewhere[39,40].

Data quality control

The VCF files were split at multi-allelic sites and left-normalized using bcftools version 1.9. Low-quality genotypes were then converted to missing (thus keeping high quality genotypes only) using three criteria: allelic balance, coverage, and the genotype quality (GQ) measure from GATK. For the allelic balance, genotypes were set to missing for heterozygous calls if the ratio of reads harboring the reference allele to the total reads was outside the interval [0.25 – 0.75] [52]. They were set to missing for homozygous reference calls if the ratio was < 0.75, and for homozygous alternative alleles if the ratio was > 0.25. They were also set as missing when the coverage was < 5 or > 1.5 times the mean coverage (across

individuals), or if GQ was < 20. The following filtering steps were performed on the obtained data and removed:

- individuals with a heterozygotes to non-reference homozygotes ratio >3: the heterozygosity ratio as described and explored in Samuels et al. [53] and described as follows: “The heterozygosity ratio is the number of heterozygous sites in an individual divided by the number of non-reference homozygous sites and is strongly affected by the degree of genetic admixture of the population and varies across human populations. Unlike quantifications of runs of homozygosity (ROH), the heterozygosity ratio is not sensitive to the density of genotyping performed.” In our cohort, the Q-AFR group has particularly high values. As can be seen in Table 1 of the cited paper, African populations typically have the highest values and may exceed 2. Members of the Admixed group typically fall between Q-SAS and either the Persian or Gulf subgroups. Overall, the Admixed group has a higher heterozygosity ratio than the populations the admixture is drawn from, as expected.

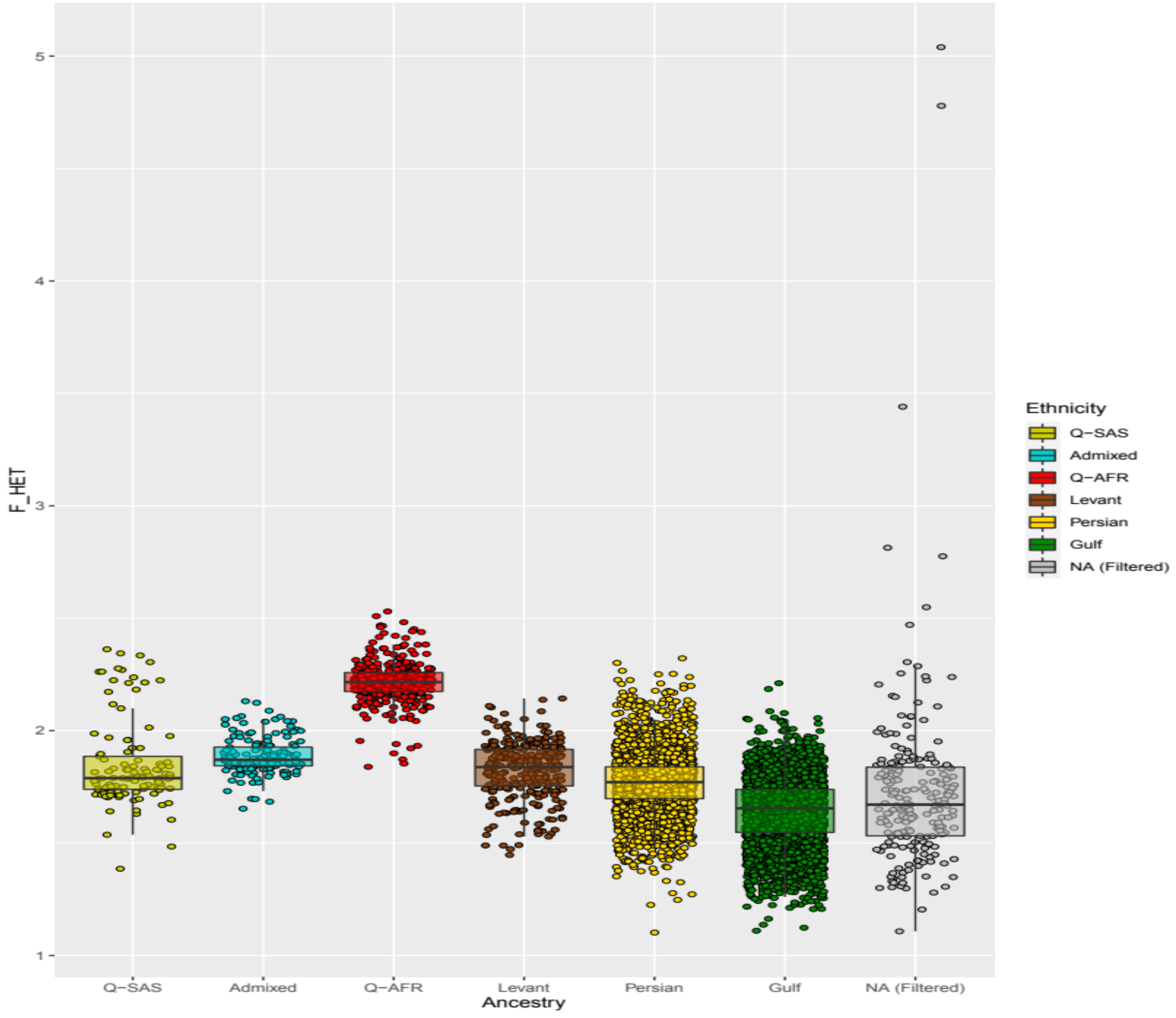

Figure: Individual heterozygosity rates (heterozygotes to non-reference homozygotes) stratified by ancestry

- variants that did not have a GATK filter value of 'PASS'
- variants with an 'ExcessHet' value >54
- variants with an 'InbreedingCoeff' < -0.2

- variants with a missing genotype rate >0.05

- individuals with missing genotype rates >0.05

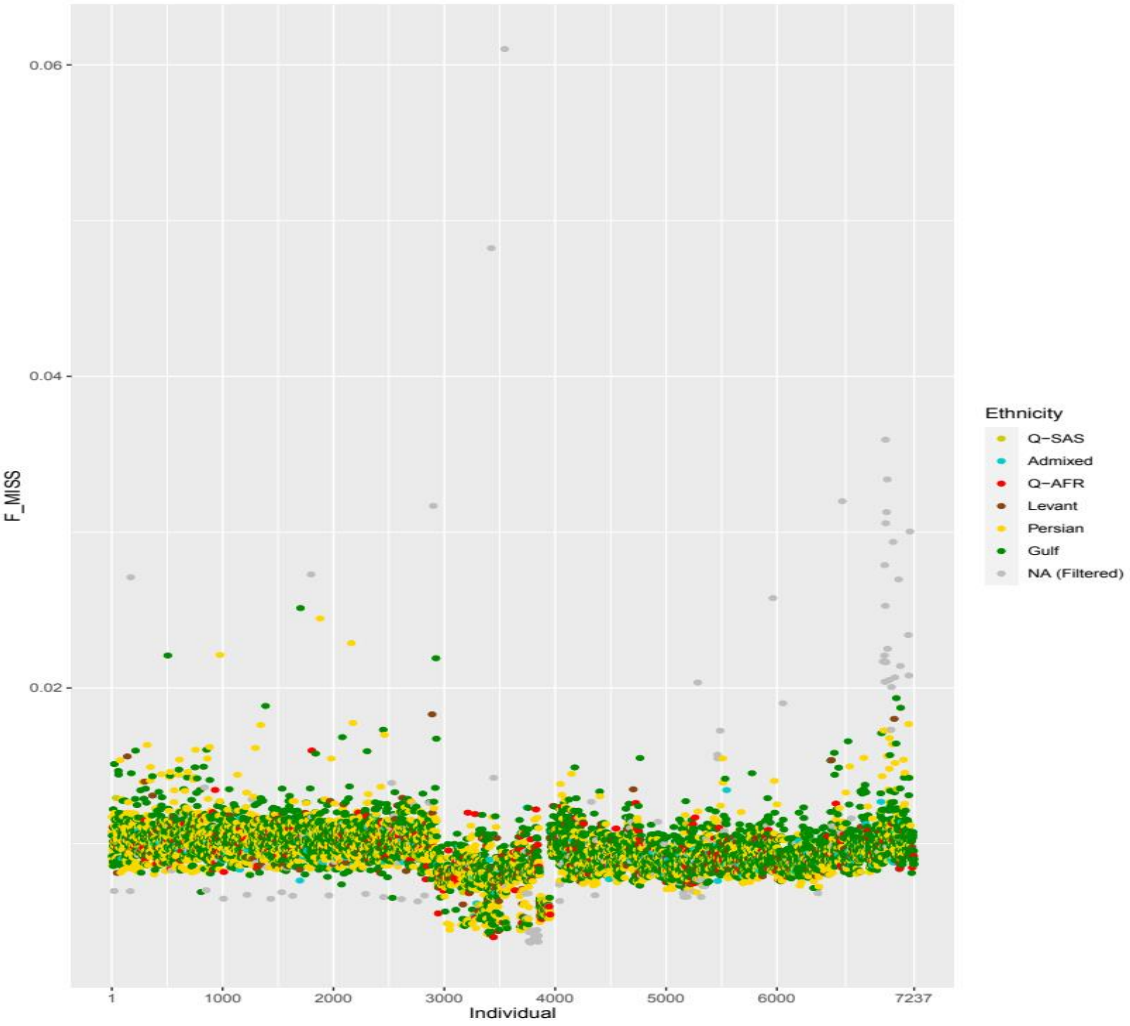

Figure: Individual missing rate stratified by ancestry

- variants that deviate from Hardy-Weinberg Equilibrium with a $P<10^{-8}$ in cases and controls combined, $P<10^{-7}$ in CHD patients, and $P<10^{-5}$ in controls, for SNVs where only observed heterozygotes were larger than expected

- individuals with ambiguous genetic sex using X-chromosome heterozygosity rate, F ($0.33<F<0.93$), estimated with PLINK v1.9 [42], The calculation of F for the X chromosome

was performed after splitting off the pseudo-autosomal region (PLINK --split-x) and then running LD-based pruning. The bounds were determined by taking a rough bound of 0.8 between the sexes (so < 0.8 considered to be female and > 0.8 considered male) because the male class shows less variation and 0.8 is a commonly used boundary for them, e.g. it is the default boundary used by PLINK for the male class. Then the final bounds were determined by using the mean of each class +/- 3 standard deviations

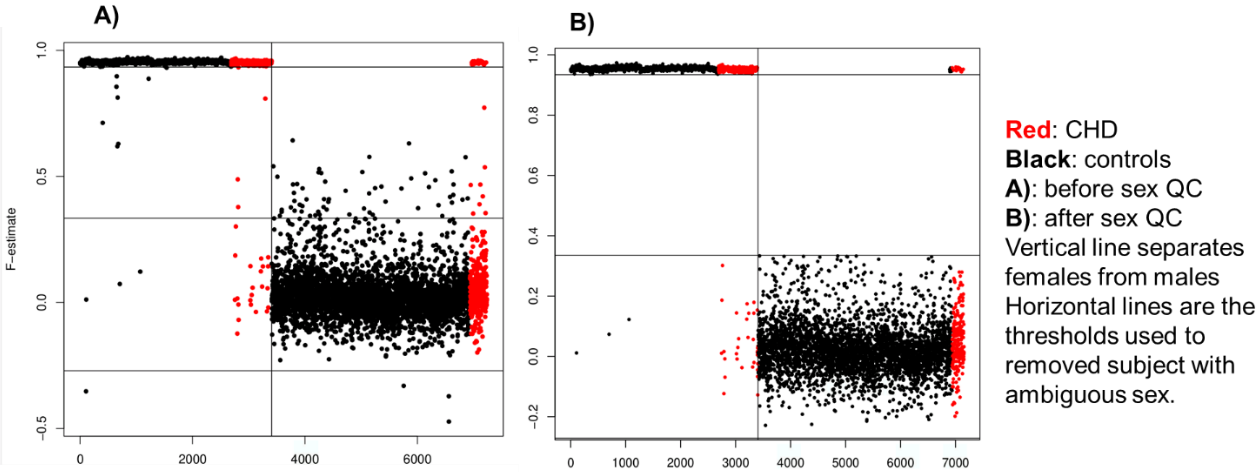

Figure: PLINK heterozygosity *F* estimate on X chromosome.

- one individual of each duplicated or highly related pair of individuals (PLINK kinship estimate > 0.4)

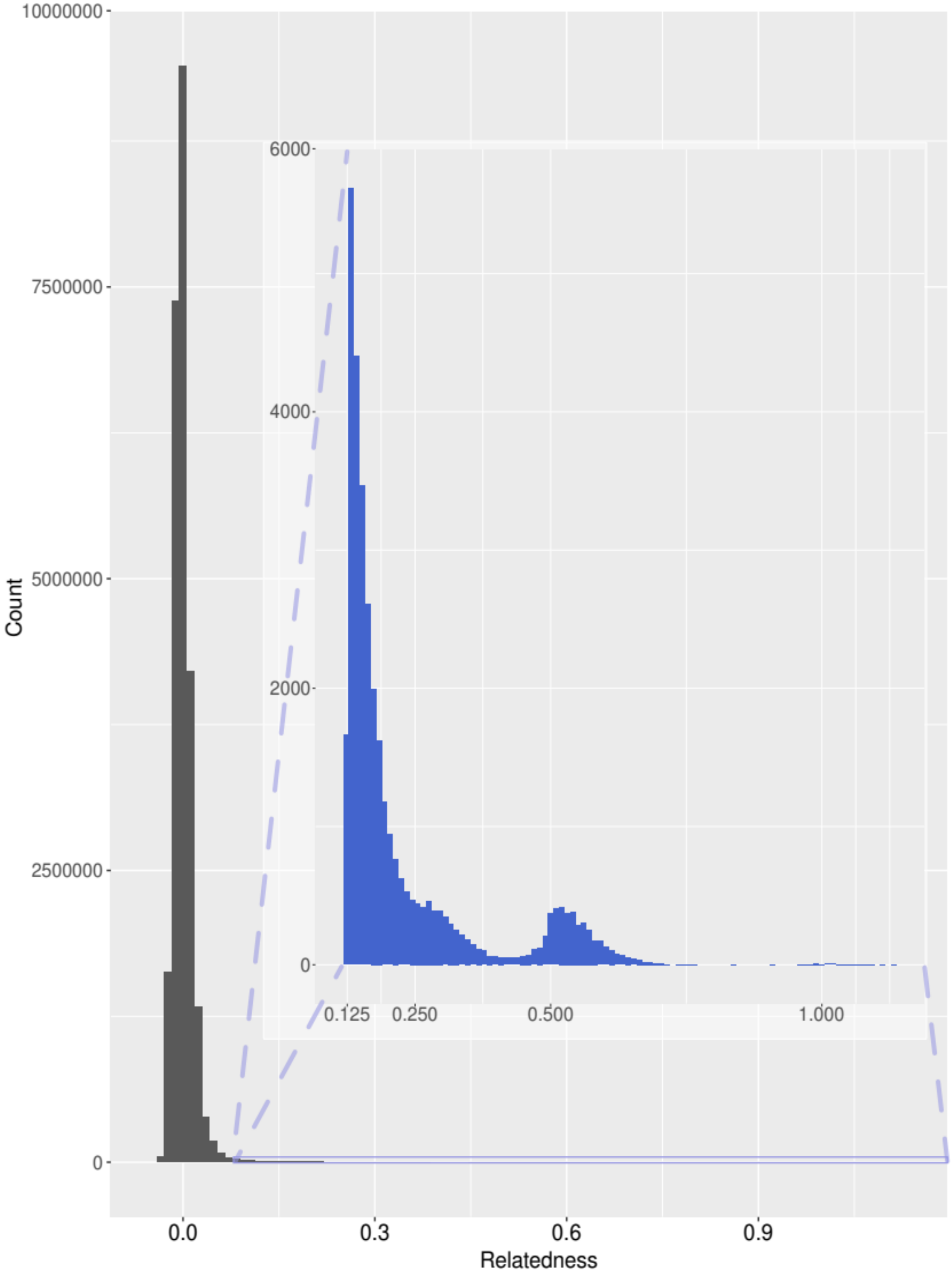

Figure: Relatedness distribution: Kinship coefficients were estimated using PLINK.

When there was a discordance between the reported sex and genetic sex, genetic sex was used. After these QC steps, 7,023 individuals remained (1,014 cases and 6,009 controls). For association analysis, autosomal biallelic SNVs that are not in low complexity regions as defined by Li H.[41] and with minor allele frequency (MAF) >0.01 were tested (8,278,389 SNVs):

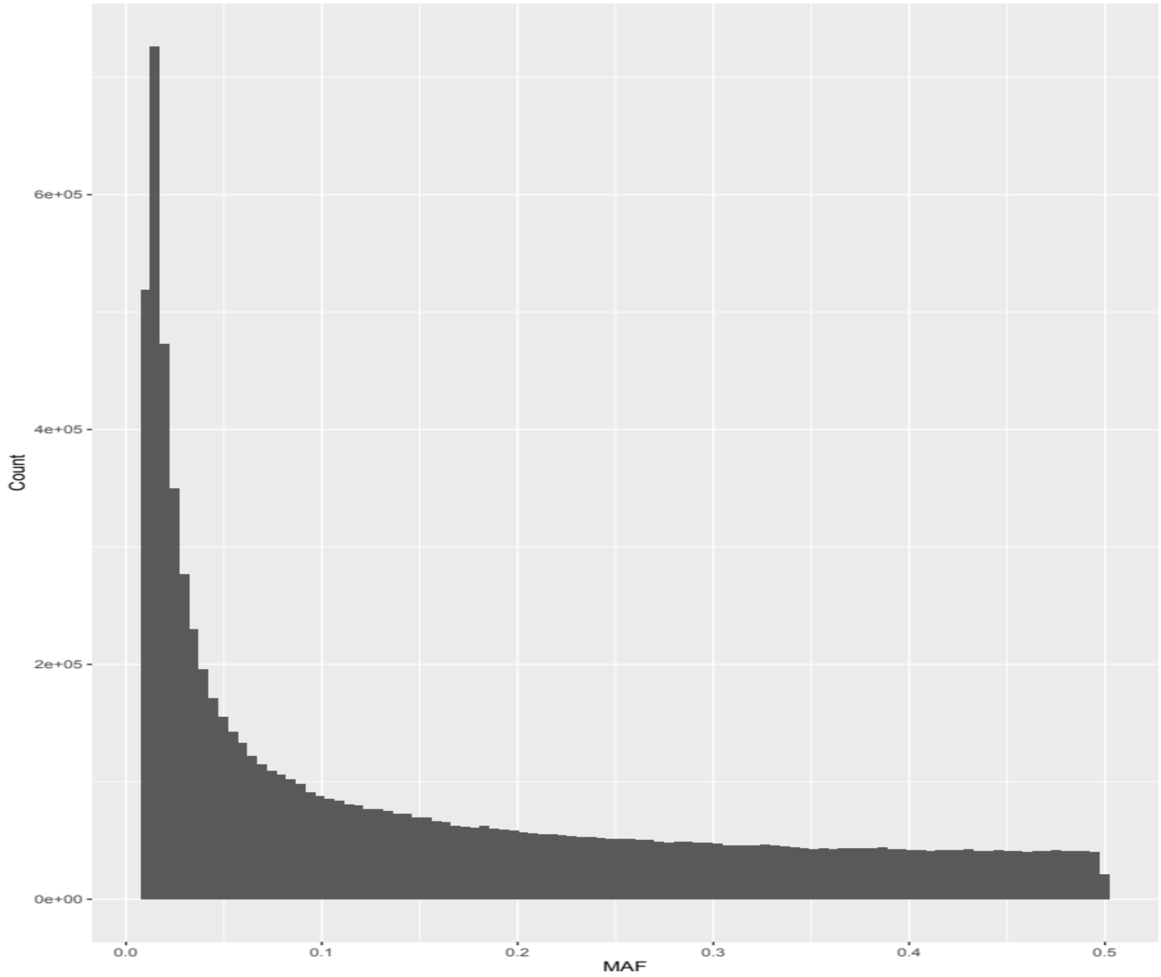

Figure: MAF distribution of the post-QC SNVs.

Statistical analysis

Relatedness estimation

Kinship coefficients were calculated to quantify relatedness between subjects using a relatively independent set of 265,250 SNVs obtained by LD-pruning SNVs with MAF >0.05. The pruning was performed using the PLINK '--indep 50 5 1.05' command. PLINK kinship coefficients were used to flag

duplicated or highly related individuals during QC steps. For association analysis, the model included the genetic relationship matrix (GRM) constructed using GCTA [43].

Association analysis for common variants

Association analysis was conducted using a generalized linear mixed model that accounts for relatedness between individuals, age, BMI, sex, and 20 PCs. Association analysis was performed using GMMAT's score test (https://cran.r-project.org/web/packages/GMMAT/index.html).[46] A Wald test was performed on the most significant SNVs to calculate their odds ratios and confidence intervals.

Known genomic loci associated with CHD

A list of previously identified CHD loci from all studied ancestry groups was extracted from the GWAS Catalog. The initial list was extracted by taking "Coronary Heart Disease" and its child traits. Only those with a "mapped trait" of "coronary heart disease" or "coronary artery disease" were retained. This list was then supplemented with lists presented in recent publications, namely McPherson et al.,[49] Erdmann et al.,[50] Charmet et al., [51] and Aragam et al.[10] The final set contained 936 unique SNVs and is presented in **Online Supplemental Material Table II**. These SNVs spanned on 400 independent loci based on LD-prunning (PLINK '--indep 50 5 1.05') conducted on the 1KG European ancestry data.

Expression quantitative trait loci (eQTL) analysis

To seek biological validation of the most significant SNVs, we performed eQTL analysis using GTEx V8.0 data in several relevant tissues ('Subcutaneous', 'Adipose Visceral Omentum', 'Artery Aorta', 'Artery Coronary', 'Artery Tibial', 'Heart Atrial Appendage', 'Heart Left Ventricle', 'Liver Muscle Skeletal', 'Pancreas', 'Small Intestine Terminal Ileum', and 'Whole Blood'; https://gtexportal.org/home/). We searched for evidence of association between SNVs and expression of neighboring genes within 1 MB.

Results

Potentially Plausible Genes

rs917306 (*CORO7/CORO7-PAM16/VASN*, *P*=1.32x10^{-6}):

CORO7 (Coronin-7), *CORO7-PAM16* readthrough, and *VASN* (Vasorin) have not been reported to be associated with CHD via GWAS previously. *VASN* has been shown to have high expression in vascular smooth muscle, to bind TGF-Beta directly (*TGFB1*, *TGFB2*, and *TGFB3*), and to modulate arterial response to injury in vivo [54]. Aging appears to reduce Vasorin expression in arterial walls and thus enhance angiotensin II signaling [55]. CORO7 codes for the protein Coronin-7 (shortname: Crn7, alternative name: 70 kDa WD repeat tumor rejection antigen homolog). Coronin-7 is widely expressed across tissue types. It is localized to the Golgi apparatus and is important for post-Golgi endocytosis. It is unique among Coronin proteins in that it is not an actin regulator, though it still interactes with F-actin. It complexes with AP-1 (Clathrin Adapter Protein 1, not Activator Protein 1) and is important for Trans-Golgi Network (TGN) transport after it is ubiquitinated at K33 to a non-degradable form by Cul3-KLHL20 Ubiquitin E3 Ligase. This then interacts with EPS-15 recruited into AP-1. NCAM-GFP and LDLR-A18-GFP were unable to leave the TGN without both Coronin-7 and KLHL20. The ubiquitinization by KLHL20 serves to recruit Coronin-7 to the TGN, and it is unneeded when it can be localized there by some other means, e.g. rapamycin [56]. LDLR is produced in the endoplasmic reticulum (ER) but are then transported to the Golgi apparatus for further modifications before they sent to the cell surface. LDLR is also recycled back to the Golgi apparatus. Mutations in CORO7-PAM could hamper the rate at which LDLR is transported from the Golgi apparatus [56]. It has been demonstrated that repression of Coronin-7 can prevent LDLR-A18-GFP from being transported away from the Golgi apparatus and that is likely also true for unmodified LDLR. *CORO7* has been found to be associated with systolic blood pressure [57] and waist-to-hip ratio [58], which are risk factors of CHD [59].

rs7690530 (*RBM47*, *P*=2.94x10^{-6}):

RBM47 is a paralog of *A1CF* and is also a cofactor for mRNA editing of ApoB (*APOB*) by APOBEC (*APOBEC1*) [60] to produce the shortened ApoB-48. ApoB, and in particular ApoB-100 or the ratio of ApoB-100 to ApoA have been considered even stronger markers for coronary atherosclerosis than LDL [61].

rs12950395 (*HS3ST3B1*, *P*=9.3x10^{-6}) and rs114906338 (*HS3ST1*, *P*=4.39x10^{-6}):

HS3ST3B1 and *HS3ST1* are involved in the synthesis of Heparan-Sulfates. Heparan-Sulfates are a major component of some tissues and are found intra and extra-cellularly as well as at the cell surface and play an essential role in many signaling pathways including TGF- Beta [62], Angiopoietin [62], and FGF [63]. *HS3ST1* may result in Heparan-Sulfate that binds Antithrombin III (*SERPINC1*), or it goes on to be processed to Heparin in Mast cells. Heparin is a potent and widely used anti-coagulant that is often still harvested from biological sources rather than produced synthetically. Two of our top SNVs (rs2480948, *P*= 2.6x10^{-5} and rs513479, *P*= 2.91x10^{-5}) are near coagulation factors 7 and 10 (*F7* and *F10*). Low dose heparin itself has been effective in reducing cardiac events and deaths, and heparin and dextran-sulfates are effective in lowering LDL and total cholesterol respectively according to Gustafsen et al. [64]. In Smits et al. [65], they observed an association between a SNV in *HS3ST1* and the severity of coronary artery disease and atherosclerotic cardiovascular events.

rs6959554 (*TNS3*, *P*=1.56x10^{-6}):

Tensin 3 is often found at focal adhesion sites. It is involved in the dissociation of the integrin-tensin-actin complex, cell migration, and potentially bone development. *TNS3* is known to interact with p130Cas/BCAR1[66], a gene known to be associated with coronary artery disease (rs999675, 10^{-12}) [28]. *TNS3* may also be involved in the regulation of RAC1 and RhoA[67,68], both of which are associated with coronary artery disease in the GWAS Catalog. TNS3 has a region homologous to PTEN with actin binding sites, Ia and Ib, and a focal adhesion binding site while the other end has an SH2 binding site, a

PTB domain, and another focal adhesion binding site. *TNS3* -/- mice are small and underdeveloped about 1/3 of the time and these cases died within three weeks of birth. They had potentially underdeveloped lung, guts, and intestines [69]. The authors mention the partial phenotypic similarity to Silver-Russell syndrome in humans, which is associated with maternal uniparental disomy of chromosome 7 around 5-10% of the time [70]. The syndrome has been linked to increased rates of congenital heart defects, ~5.5% [70] vs. ~1% for the general US population (https://www.cdc.gov/ncbddd/heartdefects/data.html) potentially via 11p15 ICR1 hypomethylation (associated with Silver-Russell syndrome 50-60% of the time (https://www.cdc.gov/ncbddd/heartdefects/data.html). Neither the *TNS3* -/- mice, nor people with Silver-Russell syndrome, seem to be associated with CHD. *TNS3* has been associated with pulse pressure (rs12668436, $7x10^{-13}$ [71], systolic blood pressure (rs12668436, $9x10^{-10}$ [57], heart rate in heart failure with reduced ejection fraction (rs192154334, $7x10^{-6}$ [72] and also with coronary heart disease itself (rs11763932, 1.91×10^{-4} [73] albeit significant at a cutoff of 0.05, not at genome-wide significance. It is perhaps notable that BCAR1 and TNS3 were both flagged in (separate) GWAS studies of Japanese populations.

Polygenic risk score stratified by sex and ancestry

1. Sex stratification

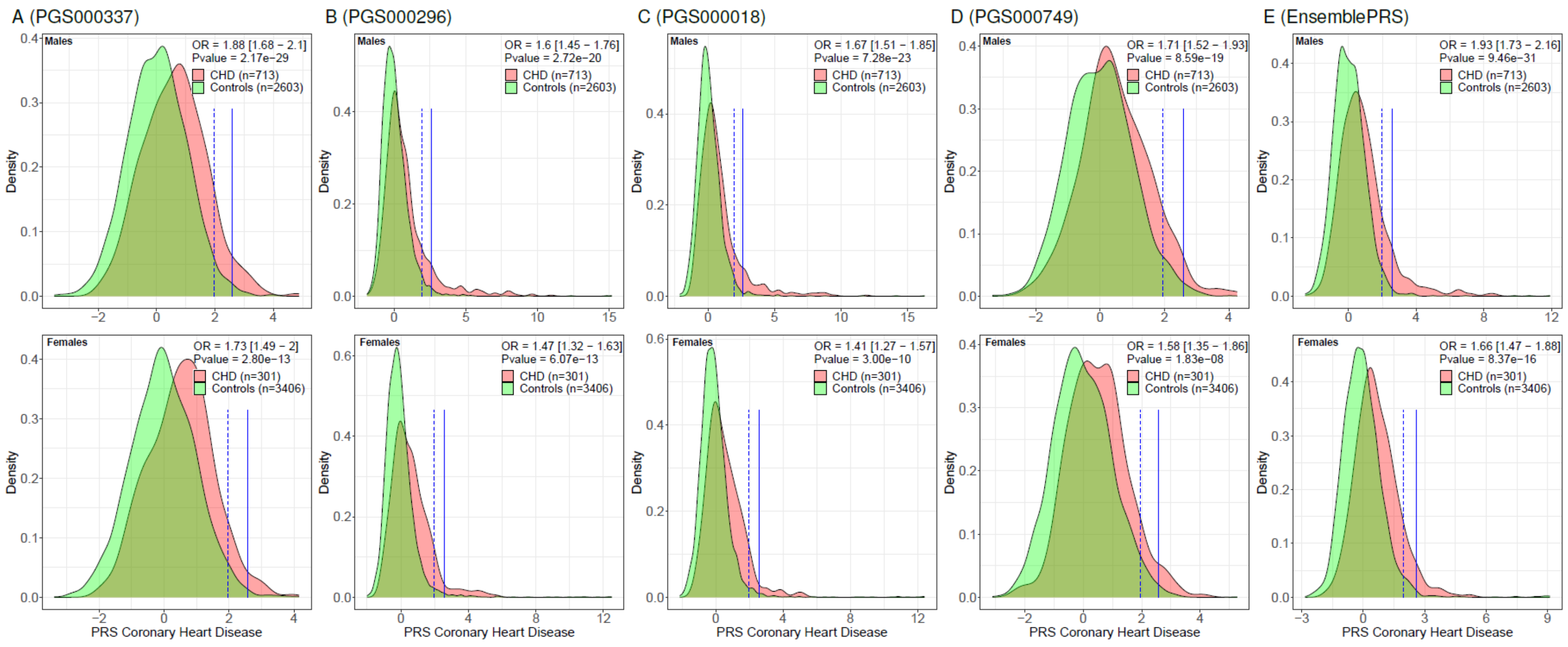

2. Ancestry stratification

PGS000337

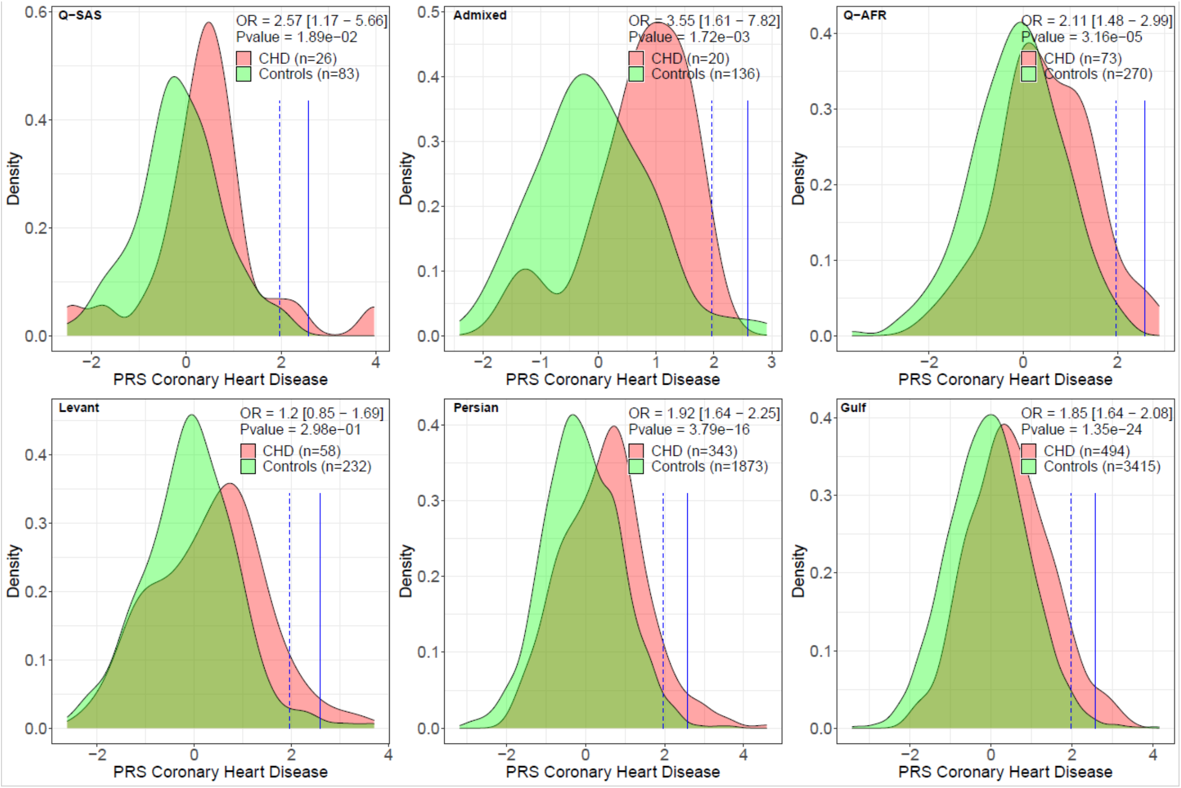

PGS000296

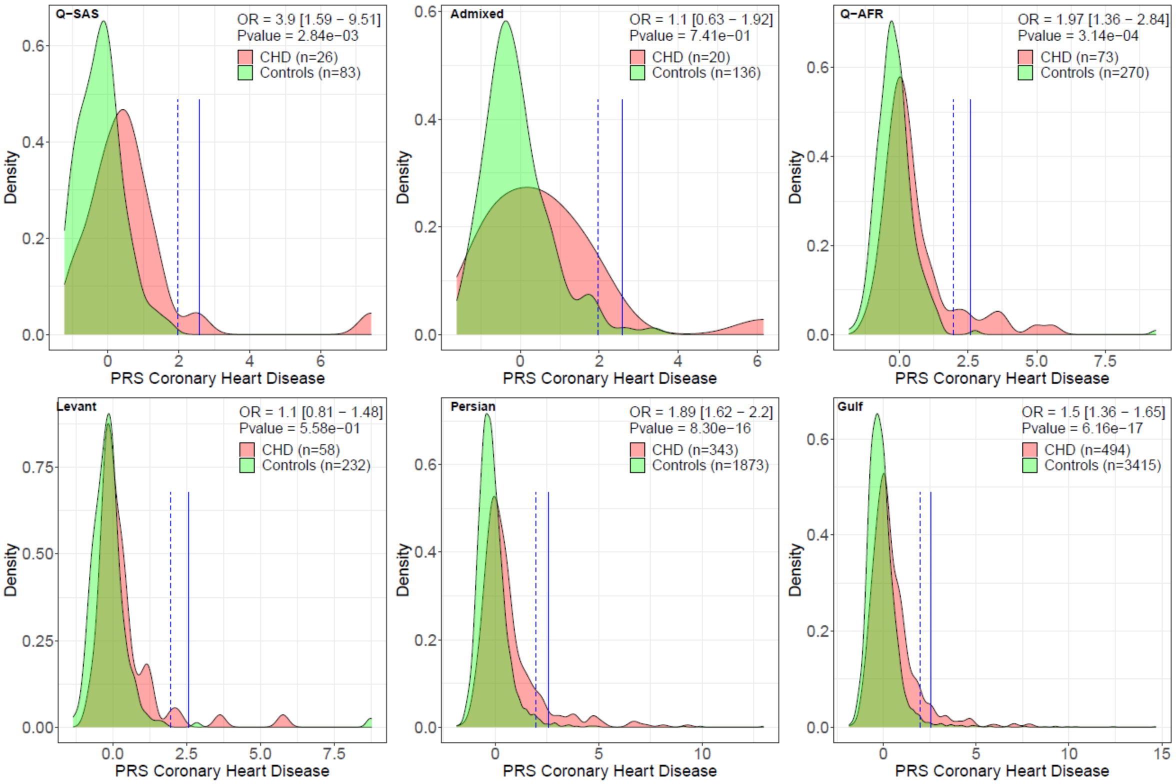
Q−SAS
OR = 3.9 [1.59 − 9.51]
Pvalue = 2.84e−03
CHD (n=26)
Controls (n=83)
Admixed
OR = 1.1 [0.63 − 1.92]
Pvalue = 7.41e−01
CHD (n=20)
Controls (n=136)
Q−AFR
OR = 1.97 [1.36 − 2.84]
Pvalue = 3.14e−04
CHD (n=73)
Controls (n=270)
Levant
OR = 1.1 [0.81 − 1.48]
Pvalue = 5.58e−01
CHD (n=58)
Controls (n=232)
Persian
OR = 1.89 [1.62 − 2.2]
Pvalue = 8.30e−16
CHD (n=343)
Controls (n=1873)
Gulf
OR = 1.5 [1.36 − 1.65]
Pvalue = 6.16e−17
CHD (n=494)
Controls (n=3415)
Density
PRS Coronary Heart Disease

PGS000018

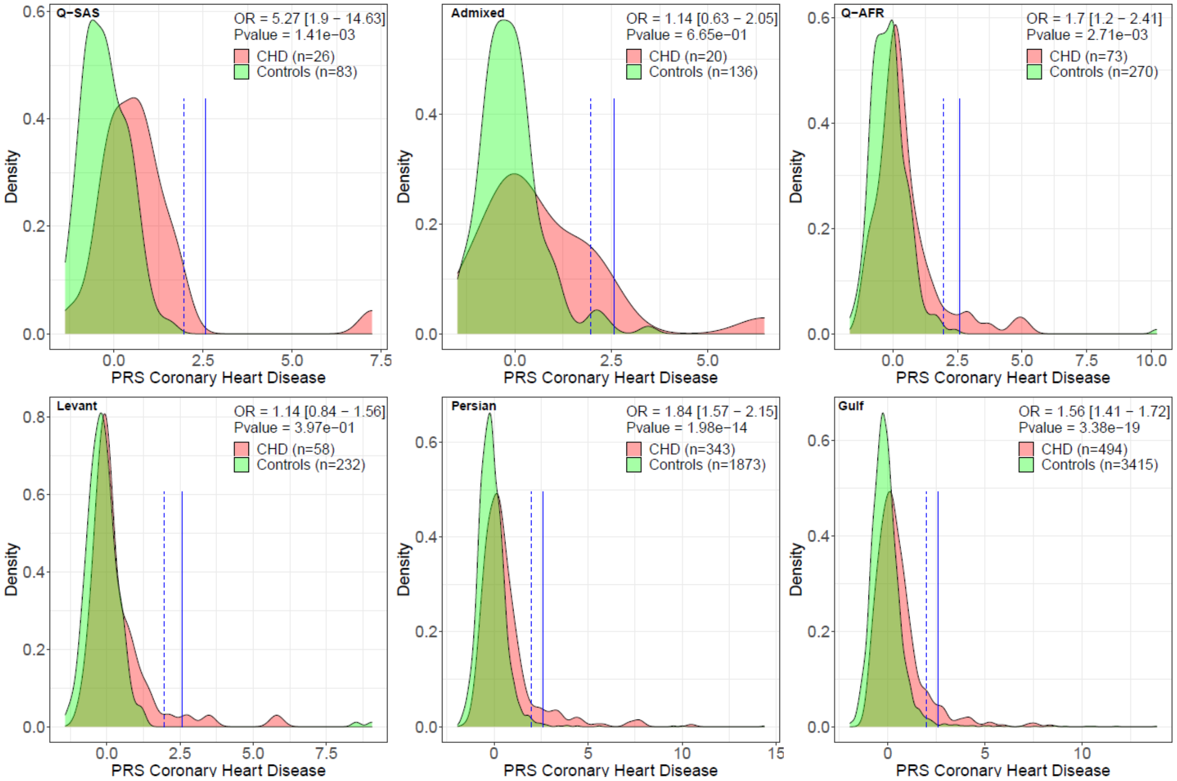

Q-SAS
OR = 5.27 [1.9 − 14.63]
Pvalue = 1.41e−03
CHD (n=26)
Controls (n=83)
Admixed
OR = 1.14 [0.63 − 2.05]
Pvalue = 6.65e−01
CHD (n=20)
Controls (n=136)
Q-AFR
OR = 1.7 [1.2 − 2.41]
Pvalue = 2.71e−03
CHD (n=73)
Controls (n=270)
Levant
OR = 1.14 [0.84 − 1.56]
Pvalue = 3.97e−01
CHD (n=58)
Controls (n=232)
Persian
OR = 1.84 [1.57 − 2.15]
Pvalue = 1.98e−14
CHD (n=343)
Controls (n=1873)
Gulf
OR = 1.56 [1.41 − 1.72]
Pvalue = 3.38e−19
CHD (n=494)
Controls (n=3415)
Density
PRS Coronary Heart Disease

PGS000749

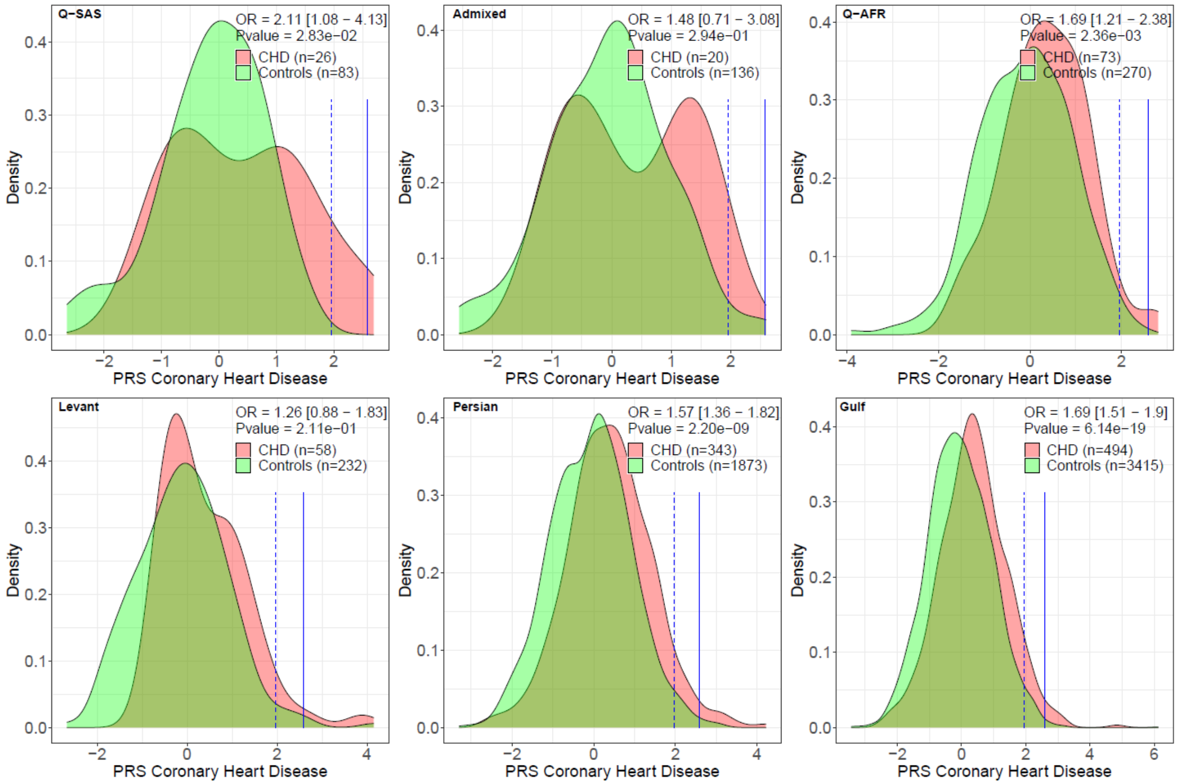
Q-SAS
OR = 2.11 [1.08 − 4.13]
Pvalue = 2.83e−02
CHD (n=26)
Controls (n=83)
Admixed
OR = 1.48 [0.71 − 3.08]
Pvalue = 2.94e−01
CHD (n=20)
Controls (n=136)
Q-AFR
OR = 1.69 [1.21 − 2.38]
Pvalue = 2.36e−03
CHD (n=73)
Controls (n=270)
Levant
OR = 1.26 [0.88 − 1.83]
Pvalue = 2.11e−01
CHD (n=58)
Controls (n=232)
Persian
OR = 1.57 [1.36 − 1.82]
Pvalue = 2.20e−09
CHD (n=343)
Controls (n=1873)
Gulf
OR = 1.69 [1.51 − 1.9]
Pvalue = 6.14e−19
CHD (n=494)
Controls (n=3415)
Density
PRS Coronary Heart Disease

EnsemblePRS

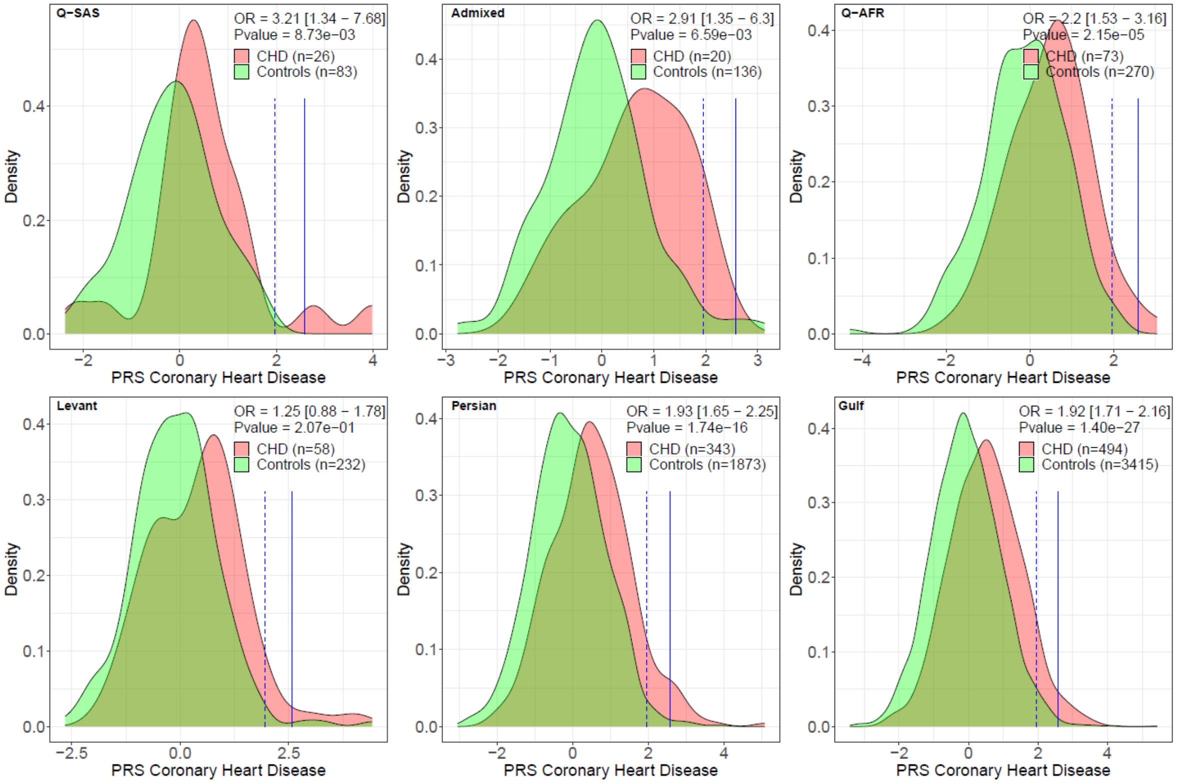

DeLong's statistical test for AUC differences

	PGS000337	PGS000296	PGS000018	PGS000749	EnsemblePRS
**PGS000337**	-	6.35E-02	3.06E-02	4.49E-03	4.72E-10
**PGS000296**	6.35E-02	-	5.59E-01	1.33E-07	1.38E-04
**PGS000018**	3.06E-02	5.59E-01	-	1.79E-07	2.34E-03
**PGS000749**	4.49E-03	1.33E-07	1.79E-07	-	6.72E-28
**EnsemblePRS**	4.72E-10	1.38E-04	2.34E-03	6.72E-28	-
